# Quantitative regulation of the thermal stability of enveloped virus vaccines by surface charge engineering to prevent the self-aggregation of attachment glycoproteins

**DOI:** 10.1371/journal.ppat.1010564

**Published:** 2022-06-09

**Authors:** Yu Shang, Li Li, Tengfei Zhang, Qingping Luo, Qingzhong Yu, Zhe Zeng, Lintao Li, Miaomiao Jia, Guoyi Tang, Sanlin Fan, Qin Lu, Wenting Zhang, Yuhan Xue, Hongling Wang, Wei Liu, Hongcai Wang, Rongrong Zhang, Chan Ding, Huabin Shao, Guoyuan Wen

**Affiliations:** 1 Institute of Animal Husbandry and Veterinary Sciences, Hubei Academy of Agricultural Sciences, Wuhan, China; 2 Key Laboratory of Prevention and Control Agents for Animal Bacteriosis (Ministry of Agriculture), Wuhan, China; 3 US National Poultry Research Center, Agricultural Research Services, United States Department of Agriculture, Athens, Georgia, United States of America; 4 Department of Avian Diseases, Shanghai Veterinary Research Institute, Chinese Academy of Agricultural Sciences, Shanghai, China; 5 Hubei Provincial Key Laboratory of Animal Pathogenic Microbiology, Wuhan, China; Chang Gung University, TAIWAN

## Abstract

The development of thermostable vaccines can relieve the bottleneck of existing vaccines caused by thermal instability and subsequent poor efficacy, which is one of the predominant reasons for the millions of deaths caused by vaccine-preventable diseases. Research into the mechanism of viral thermostability may provide strategies for developing thermostable vaccines. Using Newcastle disease virus (NDV) as model, we identified the negative surface charge of attachment glycoprotein as a novel determinant of viral thermostability. It prevented the temperature-induced aggregation of glycoprotein and subsequent detachment from virion surface. Then structural stability of virion surface was improved and virus could bind to and infect cells efficiently after heat-treatment. Employing the approach of surface charge engineering, thermal stability of NDV and influenza A virus (IAV) vaccines was successfully improved. The increase in the level of vaccine thermal stability was determined by the value-added in the negative surface charge of the attachment glycoprotein. The engineered live and inactivated vaccines could be used efficiently after storage at 37°C for at least 10 and 60 days, respectively. Thus, our results revealed a novel surface-charge-mediated link between HN protein and NDV thermostability, which could be used to design thermal stable NDV and IAV vaccines rationally.

## Author summary

The development of thermal stable vaccines can assist in the execution and expansion of global immunization programs, due to their decreased dependence on the cold chain for transport and storage. Research into the mechanism of viral thermostability may provide strategies for developing thermal stable vaccines. However, the mechanism by which an enveloped virus inherits its thermostability is poorly understood. Previously, we identified that the attachment glycoprotein (hemagglutinin-neuraminidase, HN) was responsible for the thermostability of Newcastle disease virus (NDV). In the present study, we further demonstrated that negative surface charge of HN protein positively regulated the thermostability of NDV through preventing the aggregation of HN protein. By genetically engineering charge-associated amino acid mutations onto the surface of the attachment glycoprotein of an enveloped virus vaccine strain, we remarkably improved the NDV and influenza virus vaccine’s thermal stability and potency. Based on these data, we proposed a novel surface-charge-mediated link between HN protein and NDV thermostability, and provided a novel strategy for rational design of thermal stable enveloped virus vaccines.

## Introduction

Enveloped viruses encased by a host-derived lipid layer, such as serve acute respiratory syndrome coronavirus 2, Zika virus, influenza A virus (IAV), and Newcastle disease virus (NDV), which significantly affect the health of humans and animals, also pose a considerable threat to the global economy [1–4]. The effective prevention and control of infectious diseases usually depends on worldwide vaccination strategies. For example, vaccines against measles, mumps, and influenza, have been used to immunize billions of people. However, more than 17 million people still die from infectious diseases [5]. Many of these diseases are vaccine-preventable, and deaths are therefore caused by the underuse of vaccines. One of the predominant reasons for this is that the majority of existing vaccines are sensitive to heat, and a cold chain is therefore required to maintain the quality of vaccines during transport and storage. This creates a huge burden in developing countries in terms of cost and logistics, with the cold chain constituting up to 80% of the total cost of vaccination programs [6]. Moreover, the cold chain is not always reliable for reasons such as human errors, inappropriate cold chain equipment, and power shortages [7–9]. This may lead to the rapid loss of vaccine potency and inadequate protection against disease. The situation is particularly challenging in developing and less-developed countries. It is estimated that ~50% of vaccines are discarded annually, and poor thermal stability is the main contributing factor in this wastage [10]. Therefore, improving the thermal stability of vaccines to ensure that they are partially or completely independent of the cold chain is of great importance.

New methods have been proposed that may be used to improve the thermal stability of vaccines. Firstly, thermostable formulations of vaccines were produced by optimizing the properties of the solvent (e.g., buffer, pH, and salt concentration), and by adding stabilized excipients (e.g., sucrose, serum albumin, and nonionic surfactants) [11,12]. For example, the thermostable rotavirus vaccine, licensed in India, can be stored at a temperature below 25°C for 36 months. Secondly, by integrating the biomimetic nucleating peptides onto the capsid protein of enterovirus type 71 vaccine strain, calcium phosphate mineralization was induced on viral surfaces under physiological conditions, generating a mineral exterior. This self-biomineralized vaccine could be stored at 37°C for 1 week [13]. Thirdly, structural vaccinology could guide the design of effective vaccines through stabilizing their native conformations [14–16]. By introducing cysteine residues and filling hydrophobic cavities, the engineered pre-fusion F protein of respiratory syncytial virus showed improved thermostability and superior immunogenicity when compared to the post-fusion F protein [17]. Finally, although most of the existing vaccine strains are sensitive to heat, a few are thermostable, such as NDV V4 and TS09-C strains [18,19]. After incubation at 27°C– 32°C for 14 days, the viral titer of freeze-dried V4 vaccine only reduced from 10^10.4^ to 10^9.3^ EID_50_ per vial [20]. It has been demonstrated that the thermostability of NDV was dependent on the origin of hemagglutinin-neuraminidase (HN) protein using reverse genetic technology. A recombinant thermostable NDV strain rLS-T-HN, encoding the TS09-C HN protein in the thermolabile LaSota backbone, could still induce a protective antibody response after storage at 30°C for 16 days [21].

NDV belongs to the *Avulavirus* genus in the family *Paramyxoviridae*, and encodes six structural proteins. Viral particles contain two surface glycoproteins embedded in a lipid membrane, fusion (F) protein and HN protein [22]. The HN protein is a type II integral membrane protein consisting of an N-terminal transmembrane domain (TM), a stalk region, and a C-terminal global head region. The HN is presented as a homo-tetramer with disulfide-linked dimers [23,24]. HN is a multifunctional protein involved in the recognition of sialic acid-containing receptors on the cell surface, the removal from receptors, and the interaction with F protein to promote fusion.

All proteins have surface electrostatic properties, which are strongly related to the pH of the solution and the isoelectric point (pI) of the protein itself. If the pH of the solution is equal to the protein pI, the surface charge of the protein is zero. As an intrinsic property of protein, the pI depends mostly on the dissociation constants for the ionizable groups of seven charged amino acids. Among them, Asp, Glu, Tyr, and Cys are negatively charged amino acids (NC-A), while His, Lys, and Arg are positively charged amino acids (PC-A) [25]. The surface charges are involved in a wide range of biochemical processes within the proteins, e.g., folding, binding to different targets (other proteins, nucleic acids, and the cell membrane), subcellular localization (cytoplasm, nucleus, and the cell membrane), and condensation [26]. Within the virus particles, the surface charges play an important role in various sorption processes. For example, the determination of the virus concentration from a large volume of water is achieved by adsorption to charged microporous filters [27]. The virus can be purified by chrome-focusing based on its surface charge [28].

Although the thermostability of enveloped viruses has been studied extensively [18,19,29–33], the mechanism for viral thermostability is poorly understood. In this study, using the thermostable and thermolabile NDV vaccine strains as a model, we reported a novel mechanism for the thermostability of enveloped virus. The negative surface charge of attachment glycoprotein positively regulated viral thermostability through preventing the aggregation of glycoproteins. The surface charge engineering method was thereby developed to improve the thermal stability of NDV and IAV vaccines. The surface-charge-engineered vaccines exhibited overall improved thermal stability and maintained protective efficacy even after storage at 37°C for at least 10 days.

## Materials and methods

### Ethics statement

Animal experiments were approved (Permit number: 28/2020) and supervised by the Institutional Animal Care and Use Committee of the Hubei Academy of Agricultural Sciences.

### Cells and viruses

The BHK-21 and MDCK cells were cultured in Dulbecco’s modified Eagle medium (DMEM) with 10% fetal bovine serum (FBS). The fourteen NDV strains (Ulster, TS09-C, V4, I-2, W4, D4, F48E8, 39, LaSota, Mukteswar, HN1107, HB1103, HB0901 and HB0601) and H1N1 subtype IAV strain PR8-E were maintained at Hubei Academy of Agricultural Sciences.

### Calculation of protein charges

The Isoelectric Point Calculator (IPC), a freely available, web service program (http://isoeletric.org), was used to calculate the theoretical charges of proteins at pH 7.4, based on their amino acid sequences. The 26,827 full-length sequences of attachment glycoproteins from 10 enveloped viruses were obtained from the NCBI GenBank database.

### Virus titration

The titers of viruses were determined by the HA assay in 96-well micro-plates using 0.5% chicken red blood cells, the 50% egg infectious dose (EID_50_) assay in 10-day-old SPF chicken embryos, the 50% tissue culture infectious dose (TCID_50_) assay for NDV in BHK-21 cells in the presence of 0.2 μg/ml TPCK-trypsin, and TCID_50_ assay for IAV in MDCK cells (1.0 μg/ml TPCK-trypsin) [34].

### Infectivity thermostability test

Aliquots of undiluted allantoic fluids infected with viruses were sealed in air-tight vials at 0.1 ml/vial and submerged into a water bath at 56°C for varying time points. The heat treatment was stopped by transferring the vials into an ice-cold bath. The infectivity of the virus in the vials was titrated by a TCID_50_ assay. The decreased infectivity of viruses was represented on a logarithmic scale as a function of the heat treatment time. Regression lines were plotted from four time points. Viral thermostability was shown as the mean time for a 1 log_10_ (90%) infectivity decrease (T_90_).

### Construction and rescue of mutated or chimeric viruses

For NDV mutants, the mutated infectious cDNA clones (ICs) were constructed by replacing the wild-type HN gene fragment in the backbone of the ICs with the corresponding mutated HN gene, as described previously ^[^^21^^]^. In brief, the vector fragment was PCR amplified using the backbone of the ICs as a template and vector-specific primers to exclude the HN gene fragment (6412–8262 nt). The mutated HN gene (6412–8262 nt) was constructed by multiple overlapping PCR amplifications, using the backbone of the ICs as a template and specific primers containing the corresponding mutations. Subsequently, chimeric ICs were generated by ligation of the two PCR products, i.e., the mutated HN gene and the vector fragment. Rescue of the chimeric viruses was performed by co-transfecting the mutated ICs, and the NP, P, and L supporting plasmids into BHK-21 cells pre-infected with MAV-T7. The cell lysates were collected by freeze/thawing for three times at 72 h post-transfection, passed through a 0.20-μm-pore-size filter, and inoculated directly into the allantoic cavities of 10-day-old specific-pathogen-free (SPF) chicken embryos without dilution [35].

Similarly, the chimeric NDV rTS-L-HN/A, rTS-L-HN/B and rTS-L-HN/C were constructed by replacing the HN fragments 1–126, 127–370, 371–577 aa, respectively, in ICs of TS09-C, with the counterpart fragments from LaSota strain. The chimeric NDV rLS-T-HN/A, rLS-T-HN/B, and rLS-T-HN/C were constructed by replacing the HN fragments 1–126, 127–370, 371–561 aa, respectively, in ICs of LaSota, with the counterpart fragments from TS09-C strain.

For IAV mutants, all eight gene segments of the egg-adapted H1N1 influenza virus strain A/PR/8/34 (PR8-E) were cloned into the pHW2000 vector to generate recombinant viruses as described previously ^[^^36^^]^. The construction of mutated pHW-HA was similar to that of chimeric NDV ICs. The rescue of chimeric IAVs was performed as described previously [36].

All of the rescued viruses were amplified in 10-day-old SPF chicken embryos and confirmed by a hemagglutination (HA) assay using 0.5% chicken red blood cells and DNA sequencing.

### Molecular modeling of protein structures

The structures of the attachment glycoproteins from the wild-type and mutated viruses used in this study were obtained by homology modeling using the SWISS-MODEL web server [37]. For NDV HN protein, the templates used for homology modeling were the HN structures of AV strain (PDB ID code 3T1E), Ulster strain (4FZH), and Kansas strain (1USR). For IAV HA protein, the template was the HA structure from P1/1951 strain (6N41). The protein structures were visualized using the PyMOL program (The PyMOL Molecular Graphics System, Version 1.1eval, Schrodinger, LLC). The electrostatic surface potentials and iso-potential contours of proteins were calculated and displayed using PDB2PQR and the APBS-tool of PyMOL [38].

### Virus pathogenicity assays

The pathogenicity of NDV in birds was determined by using the intracerebral pathogenicity index (ICPI) assay in 1-day-old SPF chickens and the mean death time (MDT) assay in 10-day-old SPF embryonated eggs [34].

The pathogenicity of IAV in mice was determined by inoculating the virus into six-week-old female BALB/c mice (anesthetized with ether) with a dose of 10^3.0^ EID_50_ via the intranasal route. Weight loss and survival of infected mice were monitored daily for 14 days. Mice that lost > 20% of the original body weight were humanly euthanized and documented as dead.

### Neuraminidase (NA) activity test

NA activity was tested using the NA assay kit (Beyotime, China). According to the manufacture instructions, 50 ng of HN protein or 10 μl of allantoic fluids infected with NDV was mixed with 70 μl of detection buffer, 10 μl of fluorogenic substrate and 10 μl of water. Then the mixture was incubated at 37°C for 30 min, followed by detecting the NA fluorogenic substrate cleaved by virus or protein, using a Fluorescence Spectrophotometer (Hitachi, F-7000) with an excitation wavelength of 322 nm and an emission wavelength of 450 nm. NA activity was represented as fluorescence intensity of samples above that of corresponding solutions without virus or protein.

### NDV R18 labeling and binding assay

NDV labeled with R18 was prepared as described previously [39]. Briefly, 500 μl of Hanks’s balanced salt solution (HBSS) containing the purified virus (250 μg) was mixed with 12 μl of R18 (3 μg), and incubated at room temperature for 1 hour. The unincorporated R18 was removed by filtration through a 0.22 μm syringe filter (Millipore). Finally, 500 μl of labeled virus was obtained and used freshly for the binding assay.

R18-labeled NDV were heat-treated at 56°C for 10 min, then inoculated into BHK-21 cells (moi 50) at 37°C for 30 min. Then cells were washed three times with PBS, and cultured with a complete medium. At 1 hour post infection (hpi), the cells were observed directly for the red signal of R18 by using fluorescence microscopy. At 7 hpi, the cells were fixed with 4% paraformaldehyde, stained with anti-HN rabbit antibody (prepared by our laboratory) followed by the FITC-conjugated secondary antibody (Clontech) staining, and examined for the green signal of FITC by using fluorescence microscopy.

### Western blot based NDV binding assay

Pelleted BHK-21 cells were prepared by detaching the adherent cells with versine solution and washing twice with PBS, then chilled on ice. The allantoic fluids infected with NDV were heat-treated at 56°C, then transferred to an ice-bath at the indicated time points. The heat-treated virus was divided into two portions. One was incubated with the pelleted cells (moi 10) at 4°C, and the other was kept for further western blot analysis as virus input. After incubated for 1 hour, the cells were precipitated by centrifugation, washed twice with PBS, and resuspended in DMEM of equal volume to the incubated virus. The resuspended cells binding with NDV and an equal volume of virus input were resolved by SDS-PAGE and subjected to western blot with rabbit serum against HN protein.

### Protein isolation and purification

The HN protein cleaved from viral particles of NDV was prepared as described previously [40]. NDV propagated in 10-day-old SPF chicken embryos, was purified by sucrose density gradient centrifugation. To isolate membrane proteins, the purified NDV was treated with 1% Triton X-100, and ultra-centrifugated at 100,000 g for 2 h at 4°C, then the supernatant containing membrane proteins was collected. To remove Triton X-100, the supernatant was incubated with Bio-Beads SM-2 Adsorbent (Bio-Rad), resulting in the reconstitution of virosomes containing membrane proteins. To cleave HN proteins, the virosomes were incubated with chymotrypsin (0.5 mg/ml) overnight at room temperature. The chymotrypsin digestion was terminated by adding TPCK (60 μg/ml). The cleaved HN protein was separated from virosomes by ultra-centrifugation at 100,000 rpm for 2 h at 4°C, and further concentrated by filtration through Centrifugal Filters Ultracel-50K (Millipore). The purified HN protein was analyzed by SDS-PAGE and stored at -80°C.

### Protein NA stability assay

Aliquots of purified HN protein (5.0 μg/ml) diluted with HBSS, were sealed in air-tight vials at 0.1 ml/vial. The vials were submerged into a water bath at the indicated temperature for 10 min, then transferred to an ice-cold water. The NA activities of heat-treated proteins in vials were determined by using the NA assay kit. The decreased NA activities of HN proteins were shown on a percent scale as a function of heat-treatment temperature. The protein stability of HN was shown as the temperature for a 50% decrease in NA activity of the protein heat-treated for 10 min (T_m-NA_).

### TEM images

Morphological observation of heat-treated virion (0.1 mg/ml) and purified HN (0.1 mg/ml) was carried out after negatively staining with phosphotungstic acid and uranyl formate, respectively, using a Tecani G20 microscope (FEI).

### Zeta potential and size measurement

For virion and purified HN proteins, the Zeta potentials and sizes were measured using a Zetasizer Nano ZS apparatus (Malvern). Samples were prepared at a concentration of 0.1 mg/ml in 10mM Tris-HCl (pH 5.5, 7.4 and 8.0) containing 20 mM NaCl. Measurements were carried out at 25°C.

### Circular dichroism (cd)

CD experiments were carried out on a CD spectropolarimeter by Applied Photophysics. Far-UV CD spectra were acquired in triplicate at 25°C in a 1 mm quartz cylindrical at a HN protein concentration of 0.1 mg/ml. Temperature-induced transition of HN protein was monitored by recording ellipticity at 222 nm as a function of temperature in 50 mM PBS (pH 7.5). Secondary structure estimation was calculated using Circular Dichroism analysis using Neural Networks (CDNN).

### Vaccine preparation and animal experiments

The live liquid vaccines were prepared by diluting the allantoic fluids infected with recombinant (r)NDVs to a final viral concentration of 10^7.0^ EID_50_/ml with Tris-HCl (pH 7.8) containing 3% gelatin. Groups of 2-week-old SPF chickens were immunized with fresh or stored live NDV vaccines at a volume of 0.1 ml via the intranasal and intraocular (in/io) routes, and were then challenged with a lethal dose of NDV strain F48E8 at 2 weeks post-vaccination. The challenged birds were monitored daily over a 2-week period for clinical signs and mortality. Sera were collected prior to the challenge and were detected for NDV antibody using a hemagglutinin inhibition (HI) assay.

The inactivated vaccines were prepared by inactivating the allantoic fluids infected with rNDVs or rIAVs using 0.05% Beta-Propiolactone (BPL) at 37°C for 2 h, followed by diluting to 10^7.5^ EID_50_/ml with Tris-HCl (pH 7.8). BALB/c mice and SPF chickens were used to evaluate the IAV and NDV vaccines, respectively. Groups of animals were immunized with the fresh or stored vaccines at a volume of 0.5 ml via the intramuscular (im) route, and were challenged with a lethal dose of NDV strain F48E8 or IAV strain rPR8-E at 4 weeks post-vaccination. The antibody responses prior to the challenge and protection rates were determined.

## Results

### Positive correlation between the negative surface charge of HN protein and the thermostability of NDV

**Fig 1:**
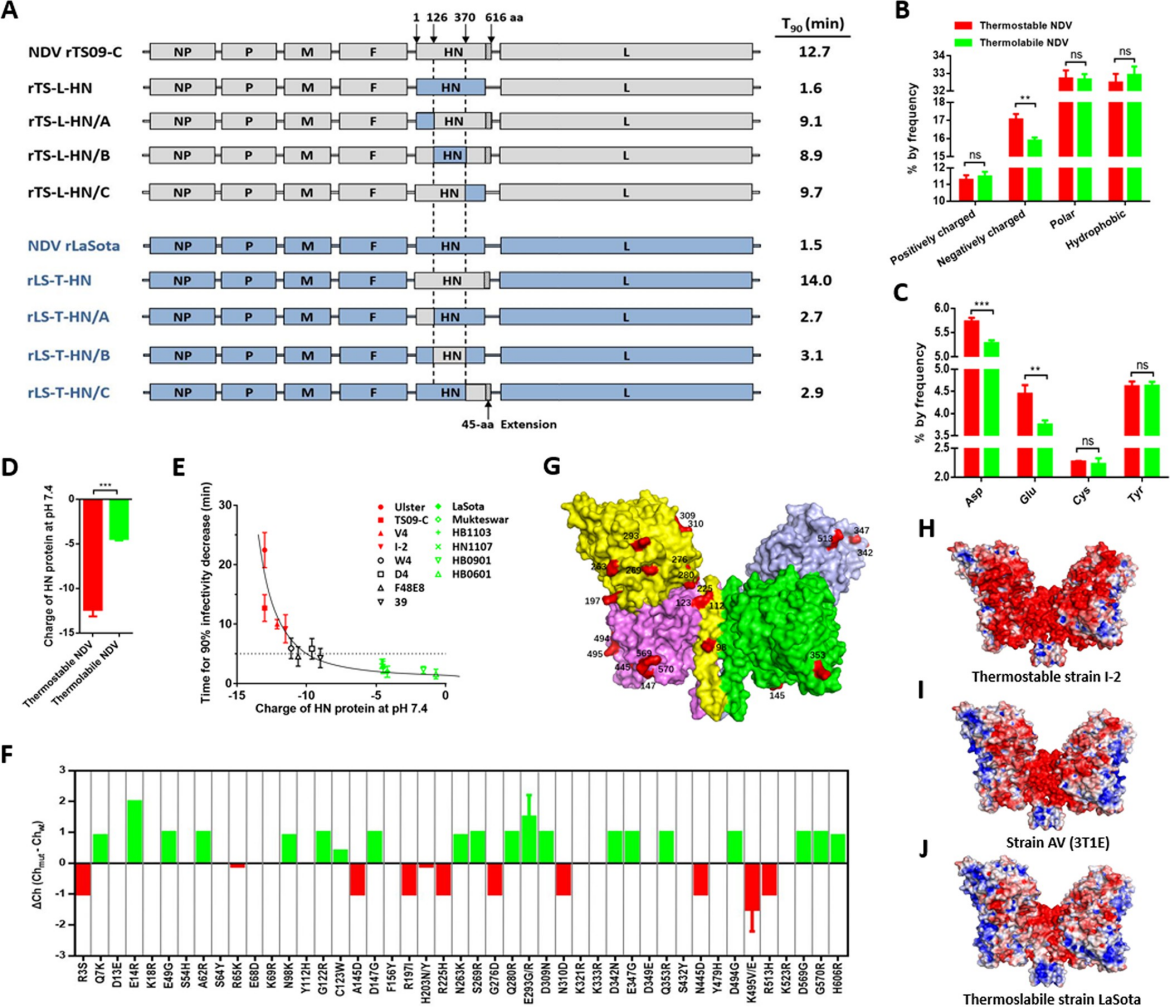
Charge of HN protein and its relationship with NDV thermostability. (A) Schematic representation showing the construction of chimeric NDVs. Grey and blue bars represent the genes of TS09-C and LaSota strain, respectively. Corresponding nucleotide numbers where the HN fragments are fused using In-fusion cloning technology are depicted. For each chimeric virus, the viral thermostability (Time for 90% infectivity loss, min) at 56°C is indicated at right side of the diagram. (B) Percentage of amino acids with different characteristic in HN proteins from thermostable and thermolabile NDV strains. (C) Negatively charged amino acids composition of HN proteins from thermostable and thermolabile NDV strains. (D) Theoretical charge of HN proteins from thermostable and thermolabile NDV strains. The four thermostable strains are TS09-C, V4, I-2, and Ulster. The four thermolabile strains are LaSota, Mukteswar, HB1103, and HN1007. The theoretical charge of protein at pH 7.4 is determined by using IPC software (http://isoelectric.ovh.org), based on its amino acid sequence. (E) Scatter diagram showing the relationship between charge of HN protein and thermostability of NDV at 56°C. Each point represents the data from one NDV isolate. (F) Effect of each charged amino acid substitution on the charge of HN protein of NDV strain TS09-C. (G) Mapping of charge-associated amino acid substitutions onto the surface of HN protein of NDV strain TS09-C. The 23 charge-associated amino acid substitutions are labeled on the protein surface and colored red. (H-J) Molecular surfaces of thermostable and thermolabile NDV HN proteins are colored according to electrostatic potentials with a range of red (- 5.0 V) to blue (+ 5.0 V). The structures of HN proteins from NDVs are obtained by homology modeling, using the HN structure of AV strain (PBD ID code 3T1E) as a template.

To explore the mechanism for thermostability of enveloped viruses, we previously reported that the major protein that determined viral thermostability was the attachment glycoprotein, by using thermostable and thermolabile NDV strains as models [21]. Here, we narrowed down the crucial region in HN where the thermostable determinant resided. The HN gene was divided into 3 fragments (A, B, and C). Six chimeric NDVs were constructed by the exchange of HN fragments between TS09-C and LaSota strain, and their thermostability was assessed (Fig 1A). All the three chimeric viruses on the background of TS09-C exhibited the greatly decreased thermostability, compared with the rTS09-C virus. However, their thermostability was still much higher than that of rTS-L-HN, which was constructed by replacing the complete HN gene in TS09-C with that of LaSota. Similar results were obtained from the chimeric viruses on the background of LaSota strain. Results suggested that a combination of amino acids located at several regions in the HN protein determined the viral thermostability.

By comparison the HN protein sequences from four thermostable and four thermolabile NDV strains, a total of 112 amino acid substitutions was found (S1 Fig). Interestingly, when comparing the contents of amino acids with different character (including negatively charged, positively charged, uncharged polar and hydrophobic) in HN protein between thermostable and thermolabile NDV strains, only the content of negatively charged amino acids was found to be significantly different between thermostable and thermolabile NDV strains (Fig 1B). Among the negatively charged amino acids, the contents of Asp and Glu in HN from thermostable viruses were significantly higher than those from thermolabile viruses (Fig 1C). Using Isoelectric Point Calculator (IPC), the theoretical charges of the HN proteins at pH 7.4 were calculated and compared between thermostable and thermolabile viruses. All the HN proteins are negatively charged, and the average negative charge of HN from thermostable viruses was 2.8-fold higher than that from thermolabile viruses (p < 0.001) (Fig 1D). The thermostability of NDV isolates was proportional to the increasing negative charge of HN protein up to -13, at which the Ulster strain exhibited a maximum viral thermostability (Fig 1E). This phenomenon indicated a positive correlation between the negative charge of HN protein and NDV thermostability.

To characterize the amino acid substitutions that may affect the charge of HN protein, we compared the HN protein sequences from four thermostable and four thermolabile NDV strains and found a total of 45 charged amino acid substitutions (S1 Fig). The influence of each charged amino acid substitution on the HN charge of TS09-C strain was analyzed, and there were 29 amino acid substitutions that considerably changed the charge (charge-associated amino acids) of HN protein (Δ Ch ˃ 0.04 and Δ Ch < ˗0.04) (Fig 1F). By homology modeling using the HN structure of AV strain (PBD ID code 3T1E, from 124 to 615 aa) as a template, the HN structures of several NDV strains were obtained and analyzed. There were 23 charge-associated amino acid substitutions located in the region of 124–615 aa, and all of them were situated on the surface of the HN homo-tetramer (Fig 1G). The electrostatic potentials on the surface of HN proteins from thermostable and thermolabile viruses were analyzed and compared. NDV bearing HN with a higher negative surface charge, exhibited higher thermostability (Fig 1H–1J). Similar results were observed by homology-modeling of these HN structures using the HN structures of strains Ulster (PBD ID code 4FZH) and Kansas (PBD ID code 1USR) as a template (S2 Fig). These data suggested that the thermostability of NDV positively correlates with the negative surface charge of HN protein.

### Negative surface charge of HN protein is the crucial thermostable determinant of NDV

**Fig 2:**
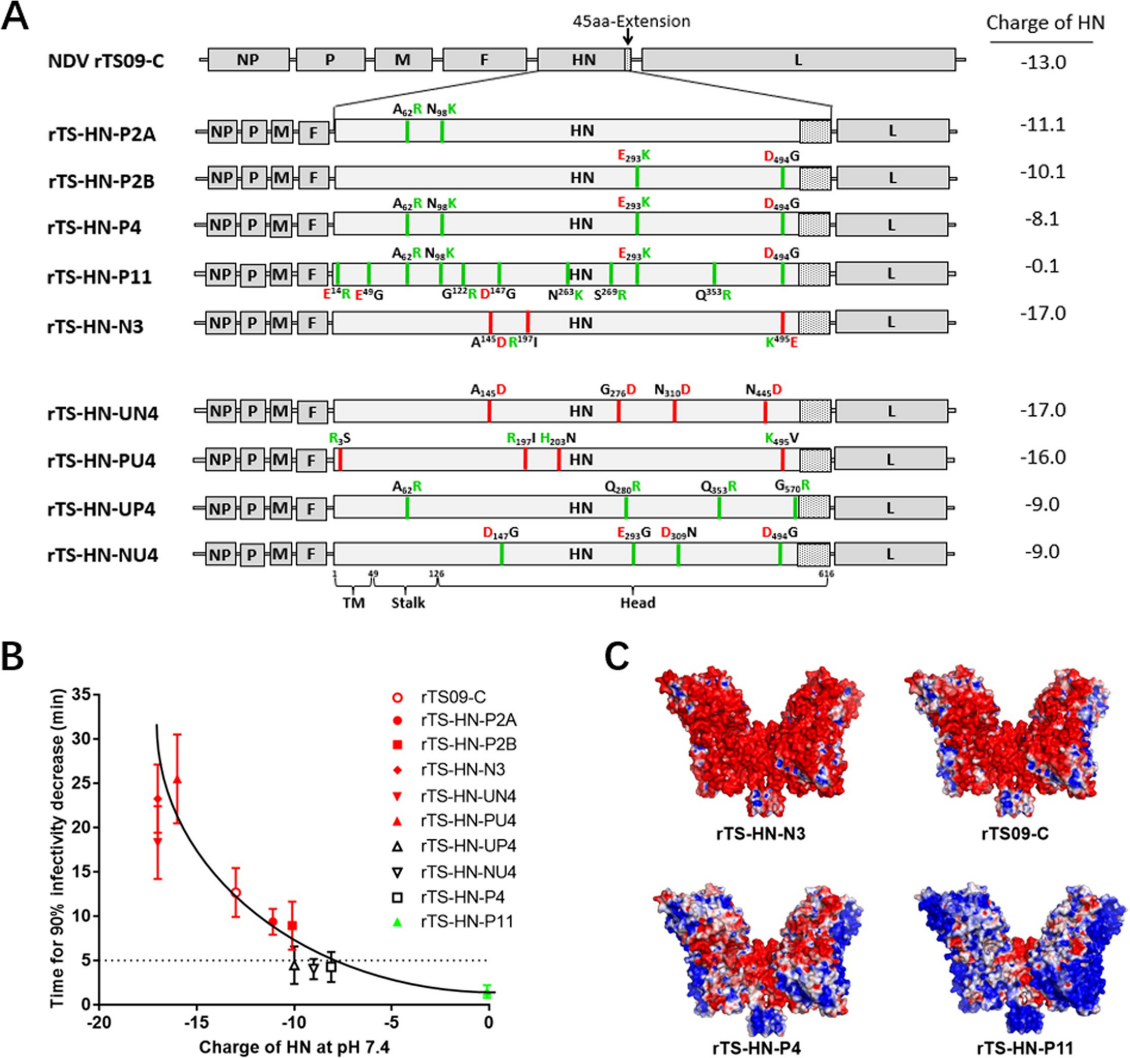
Effect of charge-associated amino acid mutations on the surface charge of NDV HN protein and viral thermostability. (A) Schematic representation showing the construction of recombinant NDVs mutated in HN proteins. In the HN protein of NDV mutants, the red and green lines indicate the amino acid mutations lead to the increase and decrease of the negative charge of HN protein, respectively. The uncharged, positively-charged, and negatively-charged amino acid residues are colored black, green and red, respectively. For each NDV mutant, the theoretical charge of HN protein are indicated at right side of the diagram. (B) Scatter diagram summarizes the relationship between the charge of HN protein and viral thermostability (Time for 90% infectivity loss, min) at 56°C. Each point represents the data from one NDV mutant. (C) Molecular surfaces of mutated HN proteins are colored according to electrostatic potentials with a range of red (- 5.0 V) to blue (+ 5.0 V). The names of NDV strains are indicated at the bottom of structures. The structures of mutated HN proteins are obtained by homology modeling, using the HN structure of AV strain (PBD ID code 3T1E) as a template.

To confirm the positive correlation between viral thermostability and the negative surface charge of the HN protein, we generated a series of NDV TS09-C mutants bearing HN proteins with different charges (Fig 2A), by introducing charge-associated amino acid substitutions obtained by comparing the HN proteins sequences (S1 Fig). Then the thermostability of NDV mutants was determined. As shown in Fig 2B, the thermostability of these NDV mutants was proportional to the decreasing negative charge of their HN proteins down to ˗0.1, at which rTS-HN-P11 had the lowest viral thermostability. NDV mutant rTS-HN-N3, with an increased negative charge of HN (Δ Ch = ˗4.0) exhibited an improved thermostability (183%, 23.3/12.7). These results confirmed that the negative charge of HN protein positively regulated the thermostability of NDV.

There are four types of basic charge-associated amino acid substitutions, including uncharged amino acid (UC-A) to NC-A, NC-A to UC-A, PC-A to UC-A, and UC-A to PC-A. To evaluate the individual effect of different types of charge-associated amino acid substitutions on viral thermostability, we generated four NDV mutants, rTS-HN-UN4, rTS-HN-PU4, rTS-HN-UP4, and rTS-HN-NU4, containing one of the four types of charge-associated amino acid mutations, respectively (Fig 2A). The viral thermostabilities of rTS-HN-UN4 and rTS-HN-PU4 bearing HN proteins with increased negative charges were higher than that of the parental TS09-C strain, while those of the other two viruses were lower than that of TS09-C (Fig 2B). Therefore, all four types of basic charge-associated amino acid substitutions in the HN protein could individually affect viral thermostability.

By comparing the electrostatic potentials colored on the molecular surfaces of the mutated HN proteins and their corresponding viral thermostability, it was also concluded that the negative surface charge of HN protein was positively correlated with the thermostability of NDV (Fig 2C). All of the NDV mutants showed similar growth titers and pathogenicity compared with their parental viruses (S1 Table). The thermostability of NDV mutants was not remarkedly changed after serial passage in chicken embryos (S3 Fig) and the revertant virus was not detected by RT-PCR sequencing assay. Taken together, our results demonstrated that the thermostability of NDV could be positively regulated by the negative surface charge of HN protein, through the method of introducing charge-associated amino acid mutations.

### Effect of negative surface charge of HN on the cell-binding stability of NDV

The main function of attachment protein is to recognize and bind to the receptors exposed on target host cells. To explore the mechanism by which the negative surface charge of attachment protein mediates viral thermostability, we evaluated the stability of the cell-binding activity of NDV mutants and their subsequent effect on viral replication in cells.

**Fig 3:**
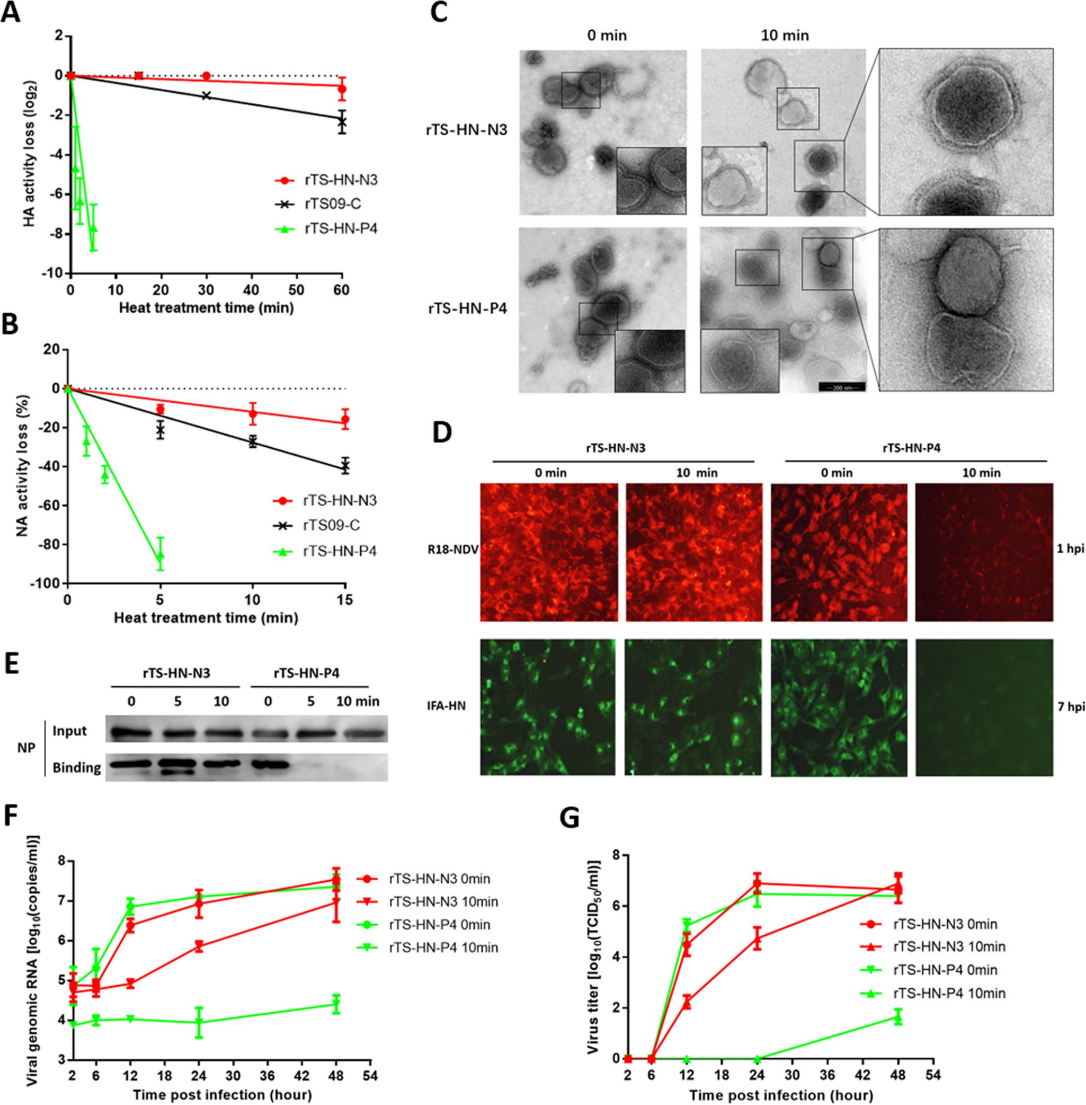
Effect of the surface charge of HN proteins on the cell-binding stability of NDV. Heat-inactivation kinetics of HA activity (A) and NA activity (B) of the NDV mutants are determined at 56°C. (C) Electron micrographs of heat-treated rNDVs. NDV mutants are heat-treated for 10 min at 56°C, then negatively stained with phosphotungstic acid. (D) R18-labeled NDV mutants are heat-treated for 10 min at 56°C, then inoculated into BHK-21 cells (moi 50) at 37°C. At 1 hpi, cells are directly observed using fluorescence microscopy. At 7 hpi, HN expression in BHK-21 cells is analyzed by IFA using rabbit serum against HN protein. (E) NDV mutants are heat-treated for the indicated times at 56°C, then incubated with BHK-21 cells (moi 10) at 4°C for 1 hour. The lysates of cells bound with NDV and equal volumes of the virus input are resolved by SDS-PAGE. Western blot is performed with rabbit serum against NP protein. (F) BHK-21 cells are infected with NDV mutants heat-treated for 10 min at 56°C (moi 1.0). Cells are harvested and lysed at the indicated time points, then analyzed for the replication level of NDV genomic RNA by using a real-time RT-PCR assay. (G) BHK-21 cells are infected with NDV mutants heat-treated for 10 min at 56°C (moi 1.0). Media from infected cells are harvested at the indicated time points and virus titers are determined by TCID_50_ titration in BHK-21 cells.

The NDV HN protein possesses HA and NA activities. Two NDV mutants, rTS-HN-N3 and rTS-HN-P4, sharing the same TS09-C backbone and representing thermostable and thermolabile strains, respectively, were examined *in vitro* for the stabilities of viral HA and NA activity. Fig 3A shows that the HA stabilities of these NDV mutants differed greatly, in the order of rTS-HN-N3 ˃ rTS09-C ˃ rTS-HN-P4. Similar results were obtained from the NA stability test (Fig 3B). These results indicated that the negative surface charge of HN protein positively regulated the HA and NA stabilities of the virus.

Morphological changes in heat-treated rNDVs were examined by transmission electron microscopy. As shown in Fig 3C, an obvious morphological difference between the heat-treated rTS-HN-N3 and rTS-HN-P4 was observed on the surface of viral particles. For rTS-HN-P4, the surface glycoproteins were completely detached from the viral envelope after heat-treatment, and only naked viral particles were observed. In contrast, the surface glycoproteins were evenly distributed on the envelope of heat-treated rTS-HN-N3. The viral particles of both rTS-HN-N3 and rTS-HN-P4 were neither aggregated nor broken by heat-treatment at 56°C. The heat-treated viruses did not display an obvious change in particle sizes compared with the untreated viruses (S4 Fig). These findings suggested that the negative surface charge of HN affected the structural stability of the virion surface.

To evaluate the cell-binding ability of heat-treated rNDVs, NDV rTS-HN-N3 and rTS-HN-P4 were labeled with the lipophilic fluorescent dye R18, producing R18-NDVs. After heat-treatment at 56°C for 10 min, the R18-NDVs were inoculated into BHK-21 cells at 37°C for 7 h, then the HN protein was detected using an indirect immunofluorescence assay. As shown in Fig 3D, at 1 h post-infection, the R18 red signal was not observed in cells infected with heat-treated rTS-HN-P4 virus. In contrast, the cells infected with heat-treated rTS-HN-N3 displayed strong red signals. Similar results were observed from the detection of HN expression in BHK-21 cells by performing an immunofluorescence assay at 7 h post-infection (Fig 3D). The cell-binding efficiency of the heat-treated virus was further evaluated by performing an immunoblot assay. After heat-treatment for 10 min, cell-binding NP protein was more abundant in rTS-HN-N3 than in rTS-HN-P4 (Fig 3E). Regarding the replication levels of the genomic RNA and infectious virus, the heat-treated rTS-HN-N3 showed slightly lower levels than the untreated virus, but these were still substantially higher than the heat-treated rTS-HN-P4 (Fig 3F and 3G). Therefore, the negative surface charge of HN affected viral thermostability mainly through regulating the cell-binding stability of the virus.

### Effect of a negative surface charge on the temperature-induced aggregation of HN protein

**Fig 4:**
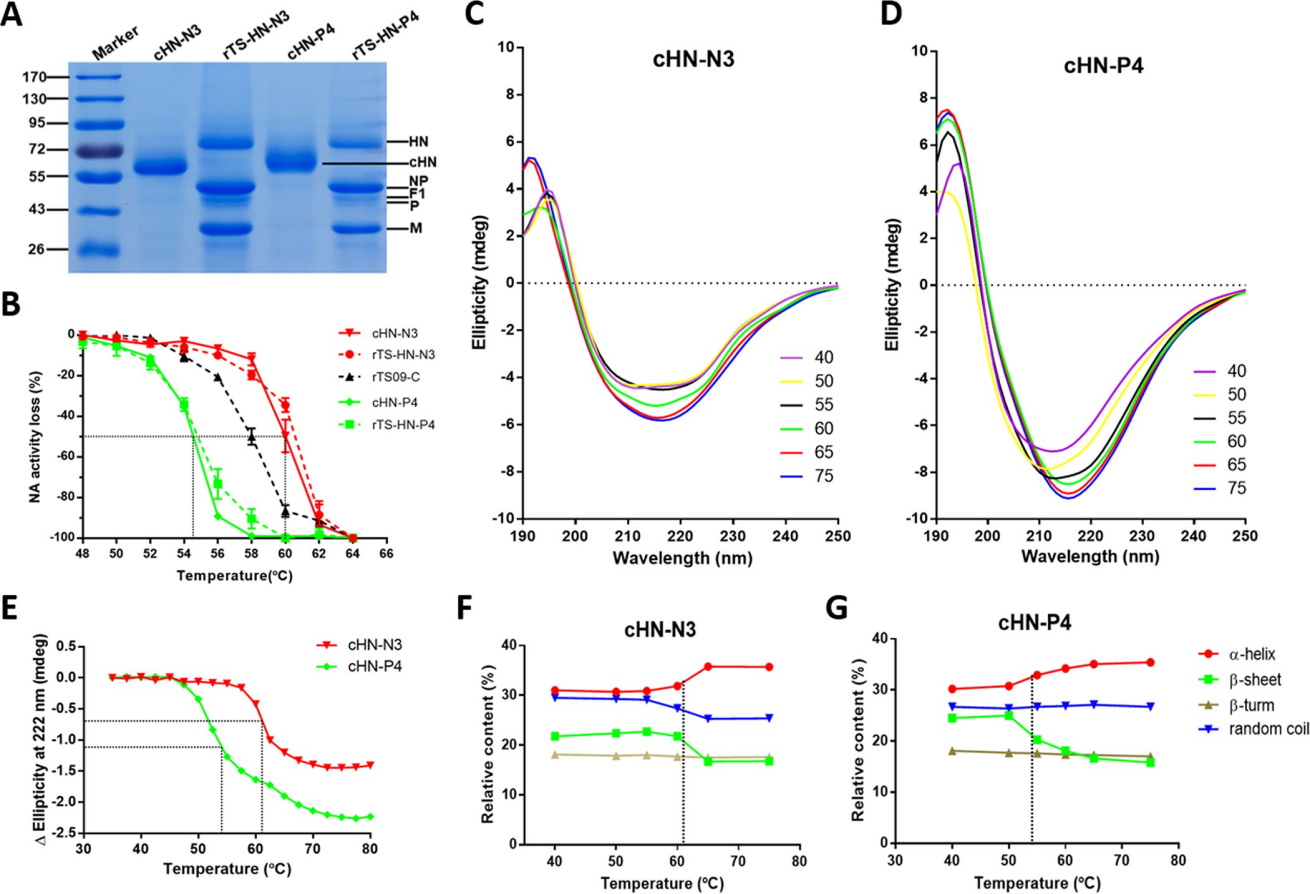
Effect of the surface charge of HN protein on its structural stability. (A) SDS-PAGE analysis of HN proteins cleaved from viral particles of NDV rTS-HN-N3 and rTS-HN-P4. (B) Heat-inactivation kinetics of NA activity of cHN proteins and NDV mutants. After heat-treatment at the indicated temperature for 10 min, the proteins and viruses are tested for their NA activity. The inactivated fractions of NA activity are represented on a percent scale as a function of heat-treatment temperature. The stability of protein/virus is shown as the temperature for a 50% decrease in NA activity of protein/virus heat-treated for 10 min (T_m-NA_). (C, D) Far UV CD thermal unfolding profiles of protein cHN-N3 (C) and cHN-P4 (D). The CD spectra at different temperature ranging from 40°C (purple) to 75°C (blue) are measured using a CD spectrophotometer J-1500 (JASCO). The legend on the right shows the line colors and their corresponding temperatures. (E) Temperature-induced transition of HN proteins as monitored by the changes in ellipticity at 222 nm. The protein stability is shown as the temperature for a 50% decrease in Δ Ellipticity at 222 nm (T_m-CD_). (F, G) Secondary structure contents of protein cHN-N3 (F) and cHN-P4 (G) under different heat-treatment temperature.

To further explore the mechanism by which the negative surface charge of HN mediates the cell-binding stability of NDV, two mutated HN proteins were isolated by cleaving HN (cHN) from the viral particles of NDV rTS-HN-N3 and rTS-HN-P4 with chymotrypsin, named cHN-N3 and cHN-P4, respectively (Figs 4A and S5). The protein stability of these cHNs was characterized *in vitro* by performing an NA stability assay. Fig 4B shows that the temperature required to achieve a 50% decrease in NA activity (T_m-NA_) of cHN-N3 was considerably higher than that of cHN-P4. The T_m-NA_ difference between these two proteins was approximately 5.5°C, which was consistent with the difference between their corresponding NDV mutants. These results demonstrated that the negative surface charge of HN could also improve the stability of the protein itself.

The structural stability of the cHN proteins was further estimated following temperature-induced transition using CD spectroscopy. We analyzed the CD spectra of cHN-N3 and cHN-P4 at various temperatures ranging from 40°C to 75°C with 5°C intervals (Fig 4C and 4D). The ellipticities at 222 nm for the two proteins were plotted against temperature, and their melting temperatures T_m-CD_ were determined (Fig 4E). The T_m-CD_ of cHN-N3 and cHN-P4 were estimated to be 54.1°C and 60.8°C, respectively, similar to the T_m-NA_ values of these two proteins (54.5°C and 60.0°C). The secondary structure contents of cHN proteins at several temperatures ranging from 40 and 75°C, were computed based on the CD spectra using CDNN. When the temperature rose, the α-helix and β-sheet contents of cHN proteins were increased and decreased (Fig 4F and 4G), respectively. This computed outcome was contrary to the results obtained from typical proteins, such as BSA (S6 Fig). Overall data obtained from the HN structural stability study suggested that the negative surface charge affected the structural stability of HN protein itself.

**Fig 5:**
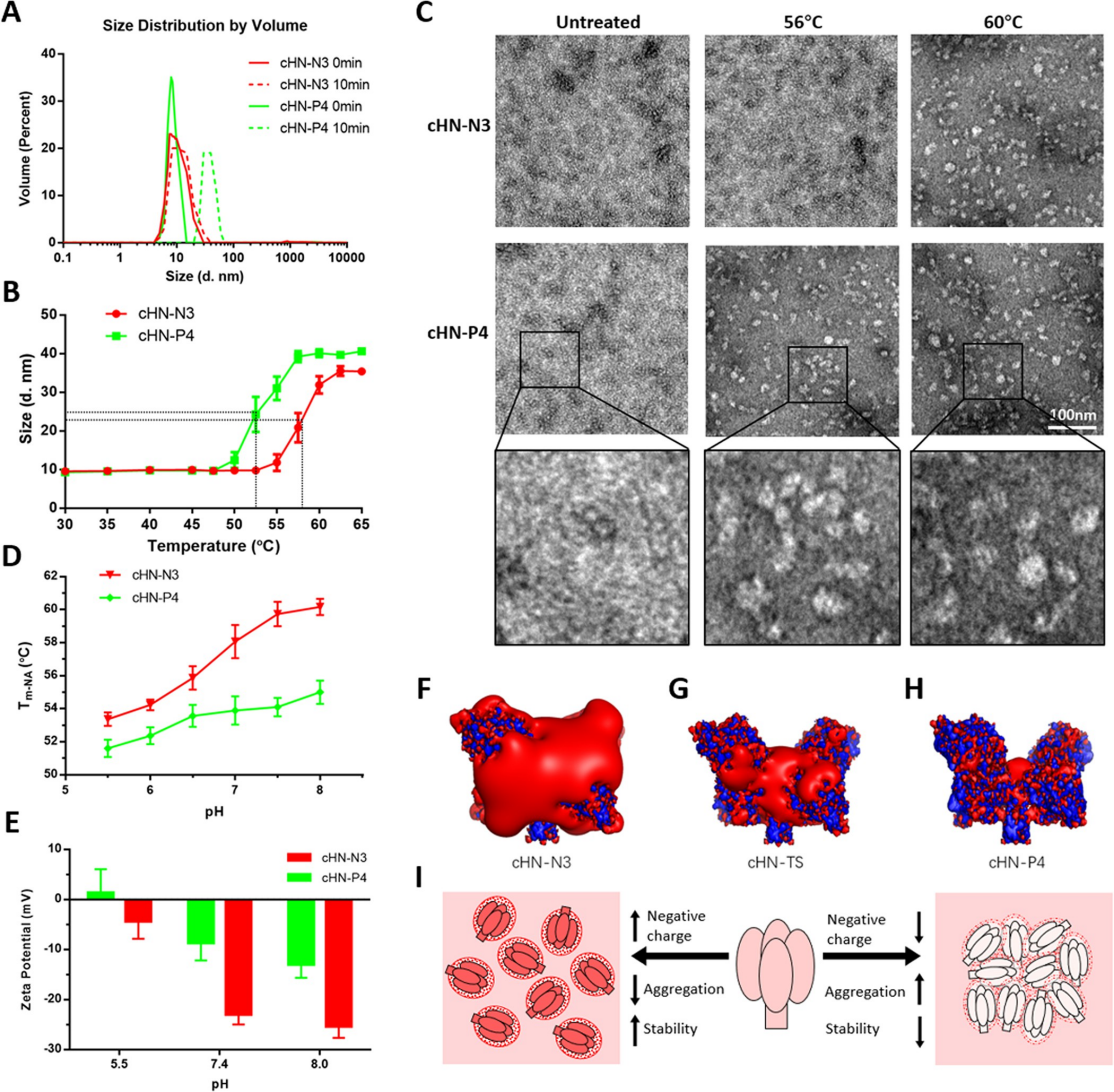
Effect of the surface charge of HN protein on its temperature-induced aggregation. (A) The DLS spectra of HN proteins heat-treated for 10 min at 56°C are measured using Zetasizer Nano ZS (Malvern). (B) Temperature-induced aggregation of cHN as monitored by protein size. After heat-treatment at the indicated temperature for 10 min, the proteins are tested for size. The protein stability is shown as the temperature for a 50% increase in the size of protein heat-treated for 10 min (T_m-SZ_). (C) Electron micrographs of heat-treated HN proteins. cHNs are heat-treated at the indicated temperature for 10 min, then negatively stained with uranyl formate. (D) Effect of pH on the stability of cHN proteins. T_m-NA_ of protein at the indicated pH is shown as the temperature for a 50% decrease in NA activity of protein heat-treated for 10 min. (E) Zeta potentials of HN proteins at the indicated pH are measured using Zetasizer Nano S (Malvern). (F-H) Negative and positive iso-potential contours of HN proteins are displayed at a level of -5 kT/e (red) and +5 kT/e (blue), respectively. The names of HN proteins are indicated at the bottom of structures. The structures of HN proteins are obtained by homology modeling, using the HN structure of AV strain (PBD ID code 3T1E) as a template. (I) Proposed model for the effect of surface charge of HN protein on its temperature-induced aggregation.

The size of cHN proteins was measured using dynamic light scattering (DLS). Under normal conditions, cHN proteins are approximately 10 nm in size. After heat-treatment at 56°C for 10 min, the size of cHN-P4 was increased to approximately 40 nm, while that of cHN-N3 remained unchanged (Fig 5A). The sizes of the two proteins were plotted against temperature, and the temperature at which a 50% increase in protein size (T_m-SZ_) was obtained was determined (Fig 5B). The T_m-SZ_ values for cHN-N3 and cHN-P4 were estimated to be 52.3°C and 58.2°C, respectively, slightly lower than the T_m-CD_ values of these two proteins. Morphological changes in heat-treated proteins were further examined by transmission electron microscopy. Unlike the untreated proteins, the cHN-P4 became aggregated after heat-treated at 56°C for 10 min, whereas cHN-N3 became aggregated only after the treatment at 60°C for 10 min (Fig 5C). These findings indicated that a negative surface charge affected the stability of HN protein through regulating temperature-induced aggregation.

The pH and ionic strength of a solution could also affect the surface charge of a protein. As shown in Figs 5D and S7, the stability of cHN proteins decreased with either decreasing pH or increasing ionic strength. The effects of both pH and ionic strength on protein stability were reversible (S8 Fig). The surface electrical charges (Zeta potentials) of the HN proteins were measured using a Zetasizer Nano ZS apparatus. As expected, the negative surface charge of cHN-N3 was increased as the pH rose, and remained consistently higher than that of cHN-P4 at the same pH (Fig 5E). The negative iso-potential contours were displayed on the surface of the cHN structures by homology modeling (Fig 5F–5H). Protein cHN-N3 displayed more marked negative iso-potential contours than those of cHN-TS and cHN-P4, in the order of cHN-N3 > cHN-TS > cHN-P4. The effect of negative surface charge on HN stability was summarized (Fig 5I). Taken together, we elucidated a mechanism for the thermostability of NDV. The highly negative surface charge of HN protein improved the structural stability of the virion surface through preventing the temperature-induced aggregation of HN and subsequent detachment from viral particles, thereby enabling the virus to efficiently bind to and infect cells after heat-treatment.

### Improved thermal stability of NDV vaccines by increasing the negative surface charge of HN protein

**Fig 6:**
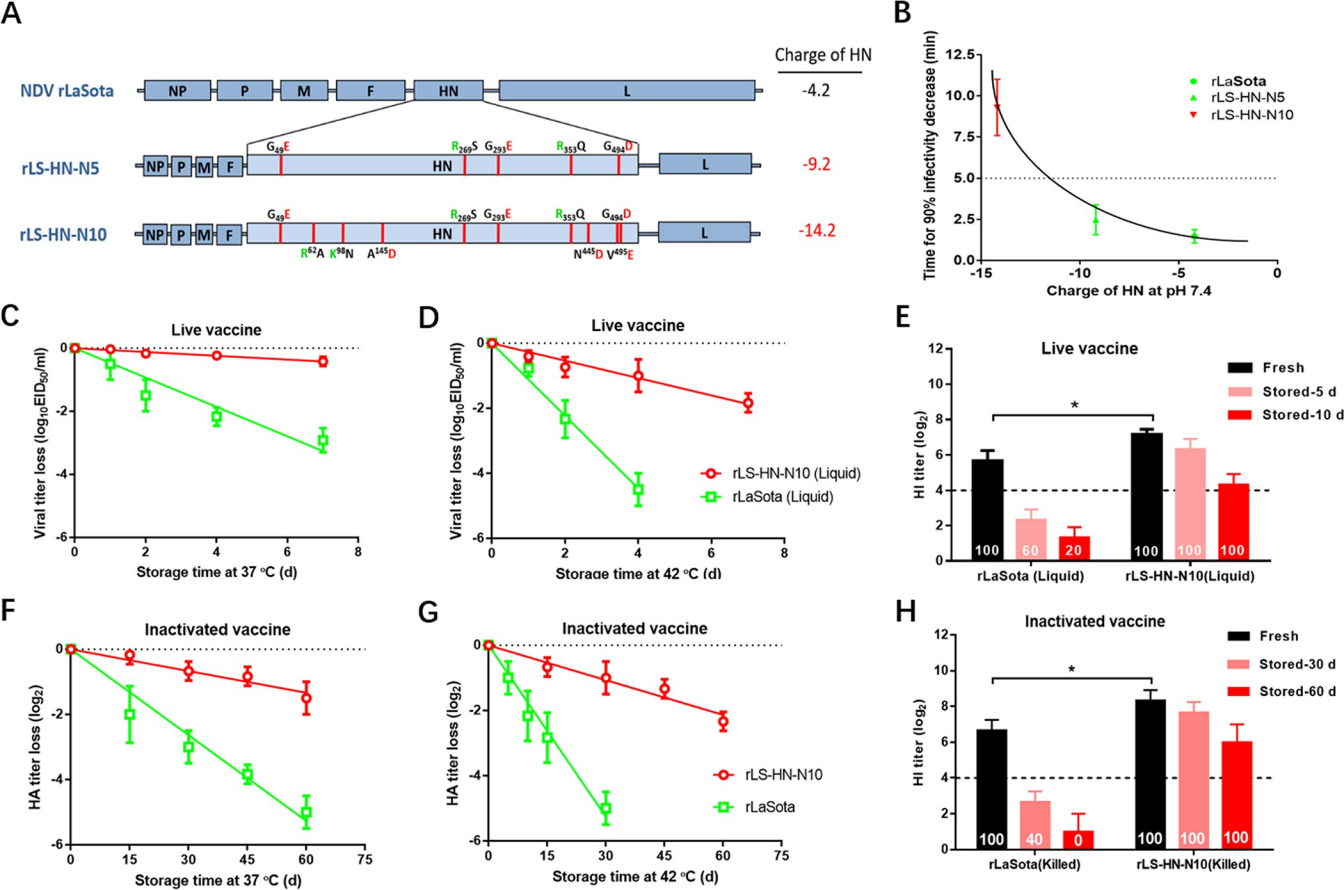
Improvement in the thermal stability of NDV LaSota vaccines by surface charge engineering. (A) Schematic representation showing the construction of recombinant NDVs with an enhanced negative charge of HN protein via introducing charge-associated amino acid mutations. (B) Scatter diagram showing the relationship between thermostability of rNDVs at 56°C and their corresponding HN charge. (C, D) Heat-inactivation kinetics of infectivity of live NDV vaccines are determined at 37°C (C) and 42°C (D). The live liquid vaccines are prepared by diluting the allantoic fluids infected with rNDVs, to a final viral concentration of 10^7.0^ EID_50_/ml with Tris-HCl (pH 7.8) containing 3% gelatin. (E) HI antibody response induced in chickens by live liquid NDV vaccines before or after storage at 37°C. The birds are immunized with the indicated vaccines at a volume of 0.1 ml, and challenged with 10^5.0^ EID_50_ NDV strain F48E8 at 2 weeks post-vaccination. The protection rates are calculated and indicated at the bottom of bar. HI titers from each group are determined prior to the challenge (n = 5). (F, G) Heat-inactivation kinetics of HA activities of inactivated NDV vaccines are determined at 37°C (F) and 42°C (G). The inactivated vaccines are prepared by inactivating the allantoic fluids infected with rNDVs by using 0.05% BPL at 37°C for 2 h, followed by diluting to 10^7.5^ EID_50_/ml with Tris-HCl (pH 7.8). (H) HI antibody response induced in chickens by inactivated NDV vaccines before or after storage at 37°C. The birds are immunized with the indicated vaccines at a volume of 0.5 ml, and challenged with 10^5.0^ EID_50_ NDV strain F48E8 at 4 weeks post-vaccination. The protection rates are calculated and indicated at the bottom of bar. HI titers from each group are determined prior to the challenge (n = 5).

Previously, the stability of NDV thermostable vaccine strain TS09-C was optimized successfully by surface charge engineering. Next, we tested whether the stability of a thermolabile NDV vaccine strain LaSota could be improved using the same method. Two NDV mutants rLS-HN-N5 and rLS-HN-N10 containing 5 and 10 charge-associated amino acid mutations in the HN protein, respectively, were constructed (Fig 6A) and tested for thermostability. Compared with the parental rLaSota strain, both NDV mutants displayed a greatly improved thermostability, in the order of rLS-HN-N10 > rLS-HN-N5 > rLaSota (Fig 6B), and retained the lentogenic pathotype and similar growth titers (S1 Table). Therefore, the thermal stability of the thermolabile LaSota strain could also be improved by surface charge engineering.

The loss of potency of rLS-HN-N10, as either a live or inactivated vaccine, during storage was evaluated. Firstly, the live liquid vaccine strains rLS-HN-N10 and rLaSota were stored at 37°C for varying time periods, and the subsequent infectivity was titrated by a TCID_50_ assay. Fig 6C shows that the T_90_ values of rLS-HN-N10 and rLaSota were 16.7 and 2.2 days, respectively. The infectivity inactivation rate of rLS-HN-N10 was 7.6-fold slower than that of rLaSota. Similar results were obtained for the live vaccines stored at 42°C (Fig 6D). The immunogenicity of the heat-treated live vaccines was then verified in animals. Live vaccines were incubated at 37°C for 5 and 10 days, and groups of birds were then immunized via the in/io routes. As shown in Fig 6E, before heat-treatment, the mean HI antibody titers in the rLS-HN-N10 and rLaSota groups were 7.2 and 5.7 log_2_, respectively. After storage for 5 days, the HI titers in the rLaSota group were considerably lower than 4.0 log_2_, and rLaSota vaccine could not completely protect birds against lethal challenge. However, rLS-HN-N10 vaccine could still provide complete protection after storage at 37°C for 10 days. Secondly, the inactivated vaccines rLS-HN-N10 and rLaSota were evaluated for potency loss at 37°C. The HA inactivation rate of rLS-HN-N10 was 4.4-fold slower than that of rLaSota (Fig 6F). Similar results were obtained for the inactivated vaccines stored at 42°C (Fig 6G). In animal experiments, after storage for 30 days, the inactivated vaccine rLaSota induced a low HI antibody response and provided only a 40% protection rate against lethal challenge. However, inactivated vaccine rLS-HN-N10 could still provide 100% protection after storage at 37°C for 60 days (Fig 6H). Collectively, these data confirmed that the thermal stability of both live and inactivated NDV vaccines could be improved by increasing the negative surface charge of HN protein.

### Improved thermal stability of IAV vaccine by increasing the negative surface charge of HA protein

**Fig 7:**
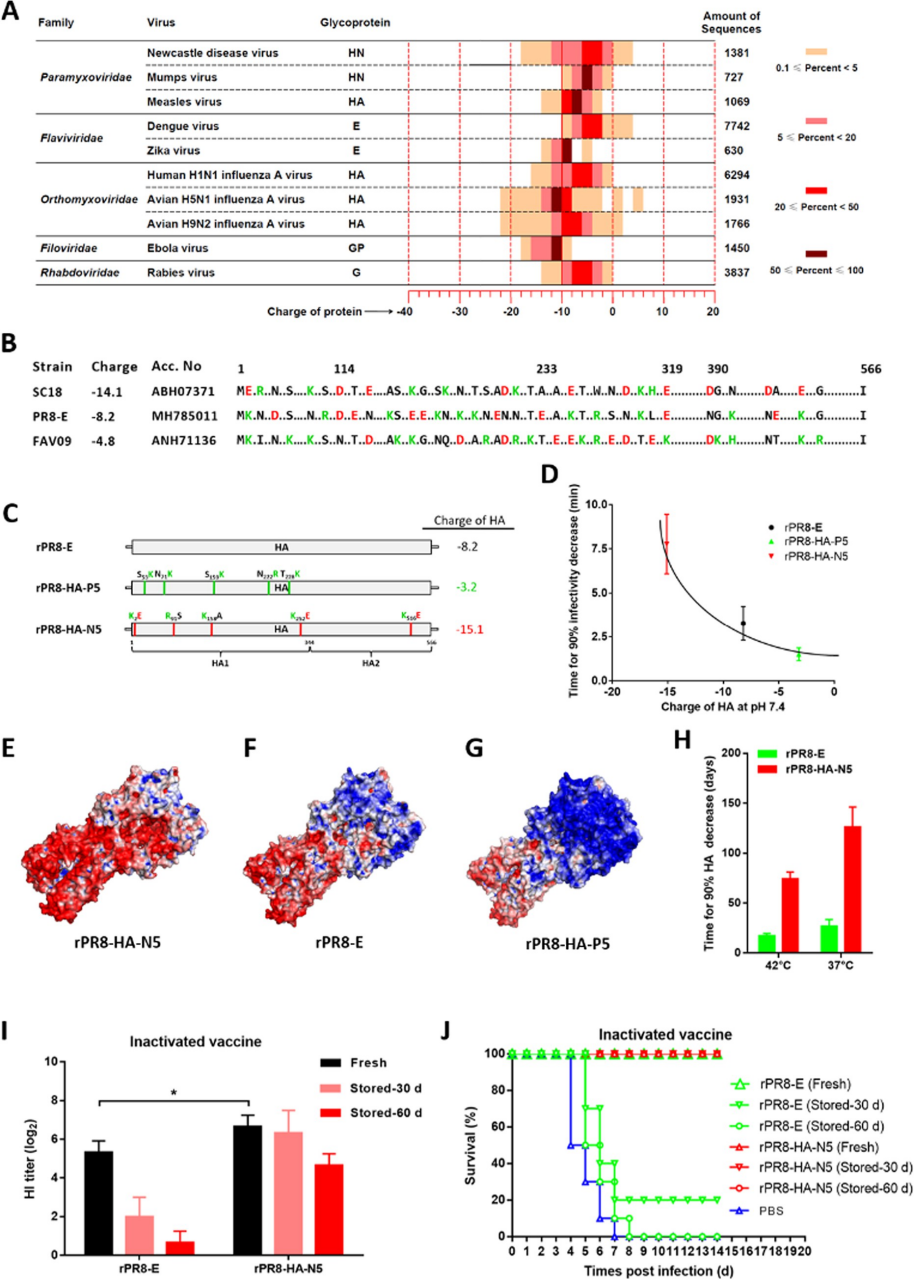
Improvement in the thermal stability of H1N1 IAV vaccine by surface charge engineering. (A) Heat map showing the percentage distribution of attachment glycoprotein sequences by charge (2.0 per unit, horizontal axis) for each enveloped virus (vertical axis). (B) Multi-sequence alignment of HA proteins from H1N1 IAV strains SC18, PR8-E, and FAV09, representing high, middle, and low negative charge at pH 7.4, respectively. The amino acid substitutions that can greatly influence the charge of HA protein are indicated with color letters. The dots represent the identical amino acid residues, or the substitutions that do not greatly influence the charge of HA protein. (C) Schematic representation showing the construction of recombinant H1N1 IAVs with changed negative charge of HA proteins via introducing charge-associated amino acid mutations. (D) Scatter diagram showing the relationship between thermostability of rIAVs at 56°C and their corresponding HA charges. (E-G) Molecular surfaces of HA proteins are colored according to electrostatic potentials with a range of red (- 5.0 V) to blue (+ 5.0 V). The names of IAV strains are indicated at the bottom of structures. The structures of mutated HA proteins are obtained by homology modeling, using the HN structure of P1/1951 strain (PBD ID code 6N41) as a template. (H) Survival of inactivated IAV vaccines. The inactivated vaccines are prepared by inactivating the allantoic fluids infected with rIAVs by using 0.05% BPL at 37°C for 2 h, followed by diluting to 10^7.5^ EID_50_/ml with Tris-HCl (pH 7.8). (I, J) Animal test of stored vaccines. The inactivated IAV vaccines are stored at 37°C for indicated times, and used to immunize BALB/c mice with a volume of 0.1 ml. At 4 weeks post-vaccination, mice are challenged with 10^3.0^ EID_50_ IAV strain rPR8-E, then monitored daily for clinical signs and mortality for 14 days. HI titers from each group (I) are determined prior to the challenge (n = 5). The percentages of survival of mice from each group (J) is calculated.

A total of 26,827 attachment glycoprotein sequences from 10 enveloped viruses were analyzed for their theoretical charges at pH 7.4. The percentage distribution of these protein sequences from each virus according to their charge was analyzed. As shown in Fig 7A, the highest negative and positive charge values of these glycoproteins were ˗22 and +6, respectively. Among these viruses, the widest charge range was observed for the HA of H5N1 IAV virus, while the HN of mumps virus, E of Zika virus, and GP of Ebola virus showed the narrowest charge range. Most of the attachment glycoproteins were negatively charged, and the charges of proteins varied greatly between enveloped viruses, even between isolates of the same virus. It was indicated that the thermostability of other enveloped viruses could also be improved by surface charge engineering.

Then we tested whether the thermostability of H1N1 IAV strain PR8-E could be improved using this method. The HA protein sequence of PR8-E was compared with those of strains SC18 and FAV09, representing relatively high and low negative charges, respectively. In total, 39 charge-associated amino acid substitutions were identified in the HA protein (Fig 7B), and 10 of them were utilized to construct two IAV mutants rPR8-HA-N5 and rPR8-HA-P5 bearing HA proteins with different charges (Fig 7C). The thermostabilities of rIAVs were assessed. As expected, the viral thermostabilities of rPR8-HA-N5 and rPR8-HA-P5 were considerably changed, in the order of rPR8-HA-N5 > rPR8-E > rPR8-HA-P5 (Fig 7D). The structures of the HA proteins from the IAV mutants were obtained by molecular modeling. Similar to the results from NDV, the IAV bearing HA with a high negative surface charge also exhibited high viral thermostability (Fig 7E–7G). In addition, two IAV mutants showed similar growth kinetics and pathogenicity compared with the parental rPR8-E virus (S9 Fig). These findings indicated that the thermostability of IAV could be regulated by surface charge engineering.

The loss of potency of rPR8-HA-N5, as an inactivated vaccine, during storage was assessed. The inactivated vaccine rPR8-HA-N5 was stored at 37°C for varying time periods, and the subsequent HA activities were determined. The mean time taken to achieve a 90% decrease in the HA activity of rPR8-HA-N5 vaccine was 126.3 days, and this was 4.7-fold higher than that of rPR8-E vaccine (Fig 7H). The immunogenicity of the heat-treated inactivated vaccines was then verified in animals. Inactivated vaccines were incubated at 37°C for 30 and 60 days, and groups of mice were then immunized via the im route. As shown in Fig 7I and 7J, before heat-treatment, the mean HI antibody titers in group rPR8-HA-N5 and rPR8-E were 6.7 and 5.3 log_2_, respectively. After storage for 30 days, the HI titer in the rPR8-E group decreased to 2.0 log_2_, and rPR8-E vaccine could provide only 20% protection against the lethal challenge. However, rPR8-HA-N5 vaccine could still provide complete protection after storage at 37°C for 60 days. Therefore, the thermal stability of IAV inactivated vaccine could also be improved by increasing the negative surface charge of HA protein.

## Discussion

**Fig 8:**
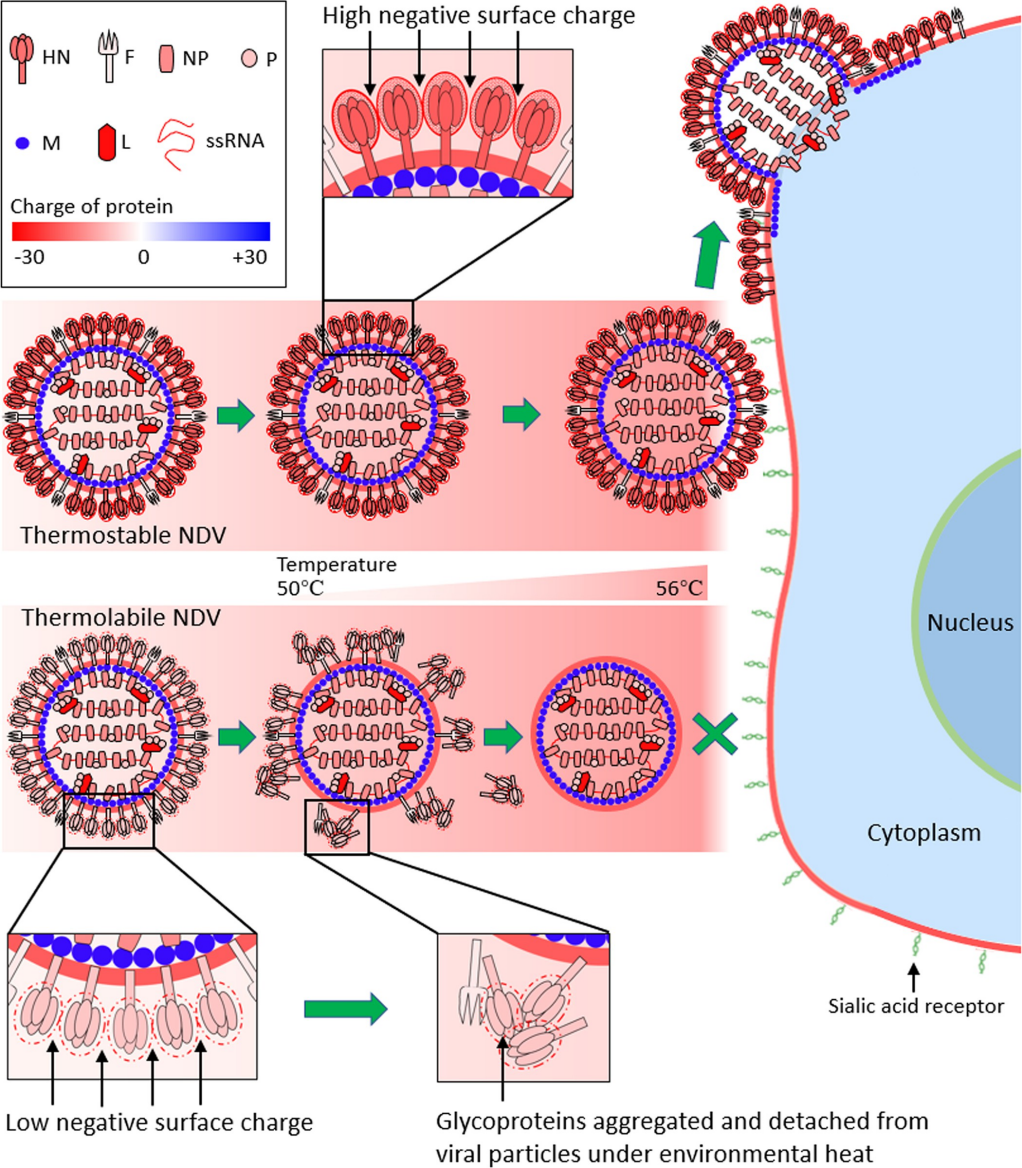
Proposed model for the surface charge of HN protein as “the key determinant” for the thermostability of NDV. The negative surface charge of HN from thermostable NDV is considerably higher than that of HN from thermolabile NDV. The first step of NDV infection is the binding of HN with the sialic acid receptor of the host cells. When thermolabile virion is subjected to environmental heat, HN proteins with low negative surface charge tend to aggregate, and detach from viral particles. Then the naked viral particles can-not bind to the cell receptors and the viral infection does not occur. However, when the thermostable virion is subjected to heat, HN proteins with high negative surface charge do not aggregate, and remain evenly distributed on the viral particles. This allows the initiation of infection and complete cycle of viral replication.

We report here a novel mechanism for the thermostability of NDV and propose its application in the rational design of thermostable NDV and IAV vaccines. The negative surface charge of HN protein positively regulated the thermostability of NDV through preventing the aggregation of HN protein. By genetically engineering charge-associated amino acid mutations onto the surface of the attachment glycoprotein of an enveloped virus vaccine strain, the thermal stability of both the live and inactivated vaccines could be greatly improved. Thus, we propose a model specifying that the surface charge of HN protein is “the key determinant” for viral thermostability (Fig 8).

The first step of NDV infection is the binding of HN protein to a sialic acid receptor on host cells. When a thermolabile virion was subjected to environmental heat, HN proteins with low negative surface charge tended to aggregate, and detach from viral particles. Then, the naked viral particles could not bind to cells and viral infection would not occur. However, when the thermostable virion was subjected to heat, HN proteins with high negative surface charge did not aggregate, and remained evenly distributed on the viral particles. This allowed for the initiation of infection and a complete cycle of viral replication. The findings that viral thermostability is regulated by the surface charge of HN protein and the potential application of this knowledge in the rational design of thermostable vaccines, highlights the importance of protein charge in the biological characterization of enveloped viruses.

The structural stability of the virion surface is of great importance in viral thermostability. For non-enveloped viruses, the outermost covering of viral particles is the capsid, which is composed of proteins. The thermostability of non-enveloped viruses is affected mainly by capsid proteins. It has been reported that the thermostability of foot-and-mouth disease virus, a typical non-enveloped virus, could be improved by replacing the amino acids located near the capsid inter-subunit interface, to establish new disulfide or electrostatic interactions between subunits, which protects the capsid against thermal dissociation into pentameric subunits [16]. Unlike non-enveloped viruses, the outermost covering of enveloped viruses comprises phospholipids and proteins, which surround the capsid. Several studies had shown that the attachment glycoproteins exposed on the surface of viral particles play an important role in viral thermostability [21,41,42]. Here, we further confirmed that the negative surface charge of HN protein affected NDV thermostability through regulating the structural stability of the virion surface.

Although the thermostability of enveloped viruses has been studied extensively, the mechanism for viral thermostability remained poorly understood. In cryo-electron microscopy studies, when incubated at 40°C, Zika virus was structurally stable and remained as a smooth-surface particle [42]. By contrast, Dengue virus, another *Flavivirus* with less thermostability than Zika virus, underwent notable structural changes from smooth to bumpy-surfaced particles at an elevated temperature of 37°C [43]. However, these conformational changes of Dengue virus particles did not affect viral infectivity [44]. Using reverse genetic technology, only a few amino acid mutations in the attachment glycoprotein that affected viral thermostability were identified [41]. In Zika virus, a single amino acid mutation T267Q within the helix αB region of E protein greatly decreased viral thermostability, indicating that the hydrophobic interactions between M protein and helix αB of the protein are critical for *Flavivirus* thermostability [45]. In IAV, the recombinant virus harboring mutation E47K in the stalk region of the HA2 subunit, displayed increased resistance to high temperature treatment, suggesting that the inter-monomer salt bridge between E21 in HA1 and K47 stabilizes the trimer structure of HA [46]. Our previous study demonstrated that HN protein was the crucial determinant of viral thermostability by exchanging NDV genes between thermostable TS09-C and thermolabile LaSota strains [21]. However, we were unable to identify the region in HN that critically affected viral thermostability through fragment exchange. This implied that viral thermostability was not dependent on a single amino acid; rather, it might be determined by a combination of amino acids located at several regions in HN protein. Inspired by the contribution of electrostatics to protein behavior [47,48], we found that the thermostability of two typical enveloped viruses, NDV and IAV, could be altered by regulating the negative surface charge of attachment glycoprotein.

Modulating the surface charge had been proven to reduce aggregation and improve the stability of proteins, such as GFP and a single chain variable fragment antibody [49,50]. Here, we successfully improved the stability of a viral attachment glycoprotein HN by increasing its negative surface charge via preventing temperature-induced aggregation. It was proposed that the long-range repulsive electrostatic forces originating from the protein’s surface charge were mainly responsible for the temperature-induced aggregation [51]. The stronger the repulsive electrostatic forces of a protein, the higher the temperature required for aggregation. These forces could be manipulated by changing the protein amino acid sequence or by changing the pH or the concentration of solute molecules and thus the surface charge [52,53]. The role of the protein surface charge was supported by colloid models. A common strategy to stabilize proteins is simply to adjust the solution pH away from the protein’s isoelectric point, which confers a net charge on the protein [54]. The value of T_m-CD_ was slightly higher than that of T_m-SZ_, and similar to that of T_m-NA_, indicating that the aggregation occurred earlier than the state transition of the protein during heat-treatment. Therefore, it was proposed that the heat-treatment induced the aggregation of HN protein, which could be influenced by the repulsive electrostatic forces derived from the protein surface charge. Then, the aggregation of protein led to the burial of most protein active sites and subsequent inactivation of the protein.

Virus in suspension also tends to aggregate near to, or at, the isoelectric point, where repulsive electrostatic forces are weakest [55,56]. The pH, the type of salts, and the salt concentration of the solution could affect the charge, repulsive forces, and subsequent aggregation of the virus [57,58]. Viral aggregates had been used to explain the nonlinear nature of virus inactivation in the environment and resistance to disinfectants [59,60]. Here, we did not detect the aggregation of heat-treated thermostable and thermolabile rNDVs (Figs 3C and S4). The charges of HN proteins distributed intensively and evenly on the viral surface, differed greatly between thermostable and thermolabile NDVs, which affected the temperature-induced aggregation and detachment of adjacent HN proteins and subsequent viral infectivity (Fig 8).

Most vaccines are sensitive to heat and are thereby strictly dependent on a cold chain to maintain their quality. This presents a huge burden for developing regions lacking reliable and extensive refrigeration infrastructure. Such regions are therefore particularly vulnerable to vaccine failure, which further increases the disease burden [61]. Thermostable vaccines with reduced dependence on a cold chain could greatly extend the global coverage of vaccines by decreasing costs, ensuring potency, and reducing waste. Here, we engineered charge-associated amino acid mutations into vaccine strains by reverse genetics to increase the negative surface charge of attachment glycoprotein which, in turn, enhanced the thermal stability of both NDV and IAV vaccines. High level antibody responses could be induced in animals immunized with the live and inactivated liquid vaccines after storage at 37°C for 10 and 60 days, respectively. These thermostable vaccines could be guaranteed for use without any access to refrigeration for at least 10 days, under a wide range of ambient temperatures, such as those experienced in tropical regions without a reliable cold chain. During the transportation and storage of vaccines, deviation from the ideal storage temperature is inevitable [62], and cases of cold chain breakdown occasionally occur. Most reconstituted vaccines must be used within a few hours because of their rapid loss of potency [63]. Our results indicated that the thermostable vaccines could be provided in a liquid formulation, without the need for freeze-drying and reconstitution before injection, and survived extended exposure times even under a wide range of ambient temperatures. In addition, compared with the control vaccine, the genetically engineered vaccines displayed slightly increased immunogenicity (Figs 6E, 6H, and 7I). A possible explanation for this was that the integrity of the virion surface of the engineered virus was superior to that of the control virus.

Unlike the previously described methods, our approach of surface charge engineering could be used to quantitatively regulate the thermal stability of vaccines by changing the negative surface charge of attachment glycoprotein. The increase in the level of viral thermostability was determined by the value-added in the negative surface charge of attachment glycoprotein. Our approach concentrated only on the surface charge of attachment glycoprotein, and the charge-associated amino acid mutations used to change the protein charge could be obtained simply from sequence alignment. The thermostable vaccines could be produced simply by propagating the vaccine strains, without additional procedures, such as the addition of the stabilized excipients or the induction of vaccine bio-mineralization [13], thus allowing pharmaceutical companies to manufacture thermostable vaccines simply and economically. Importantly, the wide charge range of attachment glycoprotein from many enveloped viruses was observed, and the sequences and structures of these attachment glycoproteins have been resolved. Therefore, these viruses could readily be genetically modified, and analogous thermostability improvements achieved by surface charge engineering might be applied to more viral vaccines. In addition to live and inactivated vaccines, other types of vaccines, such as virus-like particles and subunit vaccines, might also be genetically modified to increase the protein negative surface charge and confer improved thermal stability to vaccine products.

In summary, our study of two important enveloped viruses revealed a previously unknown surface-charge-mediated link between HN protein and NDV thermostability, and proposed its application in the rational design of thermostable NDV and IAV vaccines. These findings might reshape the current understanding of the regulatory function and mechanism of the surface charge of viral proteins. The discovery of this surface-charge-mediated mechanism for viral thermostability will improve our ability to predict the pandemic potential of circulating viruses by monitoring the charge of attachment glycoprotein. It may also provide a novel strategy for the rational design of thermostable vaccines to assist in the execution and expansion of global immunization programs, especially for developing and less-developed countries.

## Supporting information

S1 Fig Structure based sequence comparison of HN proteins between thermostable and thermolabile NDV strains. The four thermostable strains are TS09-C, V4, I-2, and Ulster. The four thermolabile strains are LaSota, Mukteswar, HB1103, and HN1007. The grey regions represent the amino acid substitutions. The charged amino acid substitutions are indicated below the NDV sequences with amino acid position numbers. In these substitutions, positively and negatively charged amino acid residues are colored green and red, respectively. The locations of β-sheets and α-helices are shown above the NDV sequences.
(TIF)

S2 Fig Molecular surface of NDV HN proteins colored according to electrostatic potentials. (A-D) The crystal structure of HN protein from Ulster strain (A) is utilized as a template for homology modeling of HN structure of strain I-2, D4, and LaSota. (E-H) The HN structure of Kansas strain (E) is used as a template for homology modeling. The electrostatic potential is mapped to the protein surface as a range of color from red (- 5.0 V) to blue (+ 5.0 V). The names of NDV strains and their corresponding thermostability (Time for 90% infectivity loss, min) at 56°C are indicated at the bottom of structures. The PBD ID codes of HN structures from strain AV and Kansas are indicated in the brackets.
(TIF)

S3 Fig Thermostability of NDV mutants after serial passages in chicken embryos. NDV mutants rTS-HN-N3 and rTS-HN-P4, and their parental virus rTS09-C were serially passaged 7 times in chicken embryos, and the thermostability of each virus at 1, 4, and 7 passages was determined at 56°C.
(TIF)

S4 Fig Size of heat-treated rNDVs determined by dynamic light scattering. NDV mutants are heat-treated at 56°C for the indicated time, then the sizes of virion are measured using Zetasizer Nano ZS (Malvern).
(TIF)

S5 Fig Isolation and purification of the cleaved HN protein from viral particles of NDV rTS-HN-N3. M, protein marker; lane 1, purified NDV; lane 2, NDV treated with Triton X-100; lane 3, reconstituted virosome; lane 4, virosome treated with chymotrypsin; lane 5, purified cHN protein.
(TIF)

S6 Fig Far UV CD thermal unfolding profiles of BSA. (A) CD spectra of BSA at different temperature ranging from 35°C (purple) to 75°C (red) were measured using CD spectrophotometer J-1500 (JASCO). The legend on the right shows the line colors and their corresponding temperatures. (B) Temperature-induced transition of BSA as monitored by the changes in ellipticity at 222 nm. (C) Secondary structure contents of BSA under different heat-treatment temperature.
(TIF)

S7 Fig Effect of ionic strength on the stability of cHN proteins. T_m-NA_ of cHN protein is measured at pH 7.4 and the indicated concentration of NaCl.
(TIF)

S8 Fig Reversible effects of pH and ionic strength on the stability of cHN-N3 protein. (A) The pH of cHN-N3 solution is adjusted from 7.4 to 6.0 by adding HCl, then back to 7.4 by adding NaOH. After heat-treatment at the indicated temperature for 10 min, the proteins under the three pH conditions (7.4, decreased to 6.0, back to 7.4) are tested for NA activity. (B) The concentration of NaCl in cHN-N3 solution is adjusted from 1.0 to 200mM by adding NaCl, then back to 1.0 mM by filtration. After heat-treatment at the indicated temperature for 10 min, the proteins are tested under three ionic strength conditions (1.0mM, increased to 200mM, back to 1mM) for NA activity. The inactivated fractions of NA activity are represented on a percent scale as a function of heat-treatment temperature.
(TIF)

S9 Fig Growth kinetics and pathogenicity of IAV mutants. (A) MDCK cells are infected with 0.002 MOI of IAV mutants. At the indicated time points, media from infected cells are collected and titrated for virus yield. (B) BALB/c mice are infected intra-nasally with 10^3.0^ EID_50_ of IAV mutants, and monitored daily for survival for 14 days.
(TIF)

S1 Table Biological characteristics of NDV mutants. (DOCX)
