# Supplementary figures and images for: Quantitative regulation of the thermal stability of enveloped virus vaccines by surface charge engineering to prevent the self-aggregation of attachment glycoproteins
Source: PLoS Pathog. 2022 Jun 9;18(6):e1010564. doi: 10.1371/journal.ppat.1010564 (PMC9182686; doi:10.1371/journal.ppat.1010564)

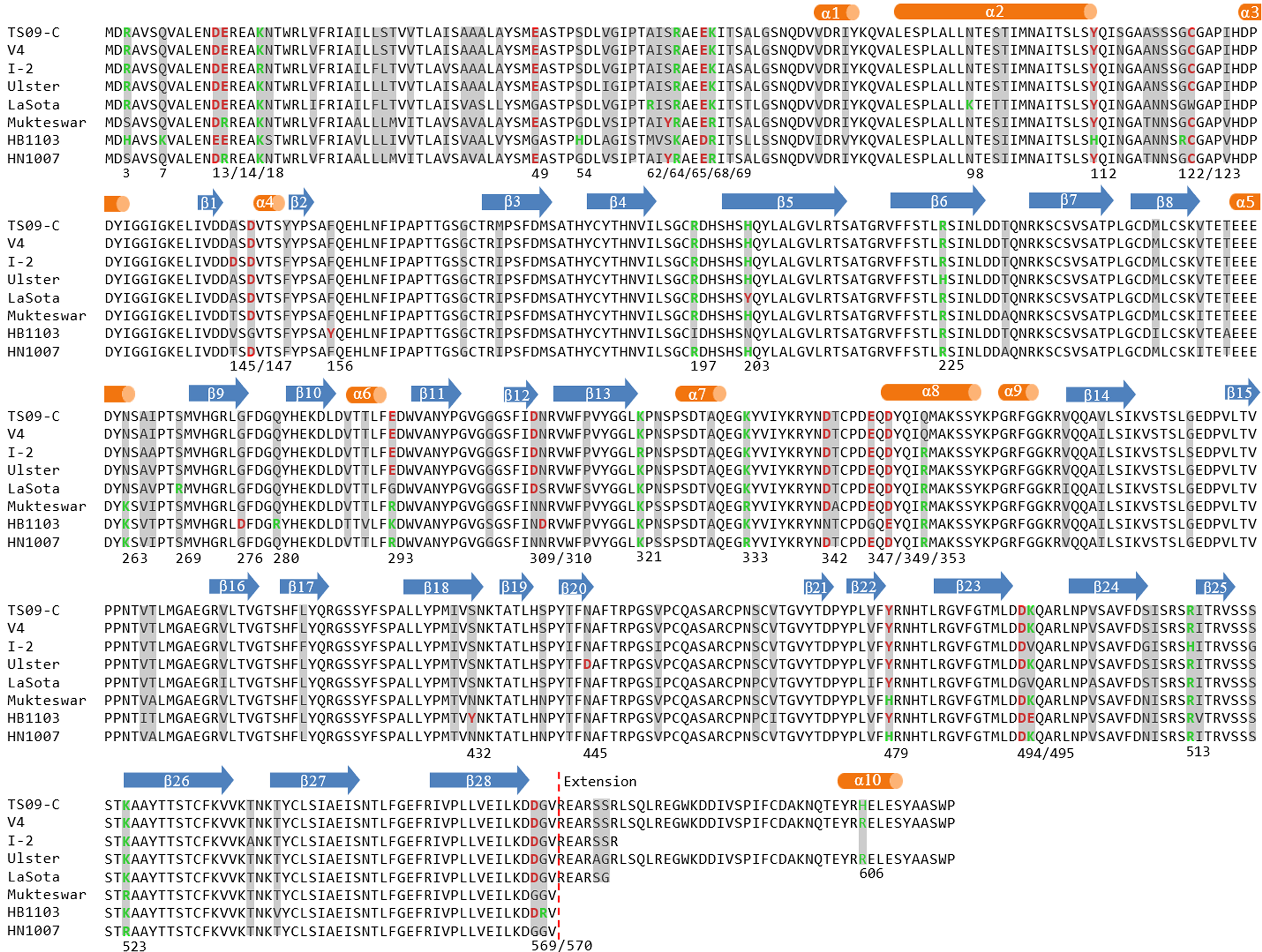

Supplement: S1 Fig — The four thermostable strains are TS09-C, V4, I-2, and Ulster. The four thermolabile strains are LaSota, Mukteswar, HB1103, and HN1007. The grey regions represent the amino acid substitutions. The charged amino acid substitutions are indicated below the NDV sequences with amino acid position numbers. In these substitutions, positively and negatively charged amino acid residues are colored green and red, respectively. The locations of β-sheets and α-helices are shown above the NDV sequences. (TIF) [file ppat.1010564.s001.tif]

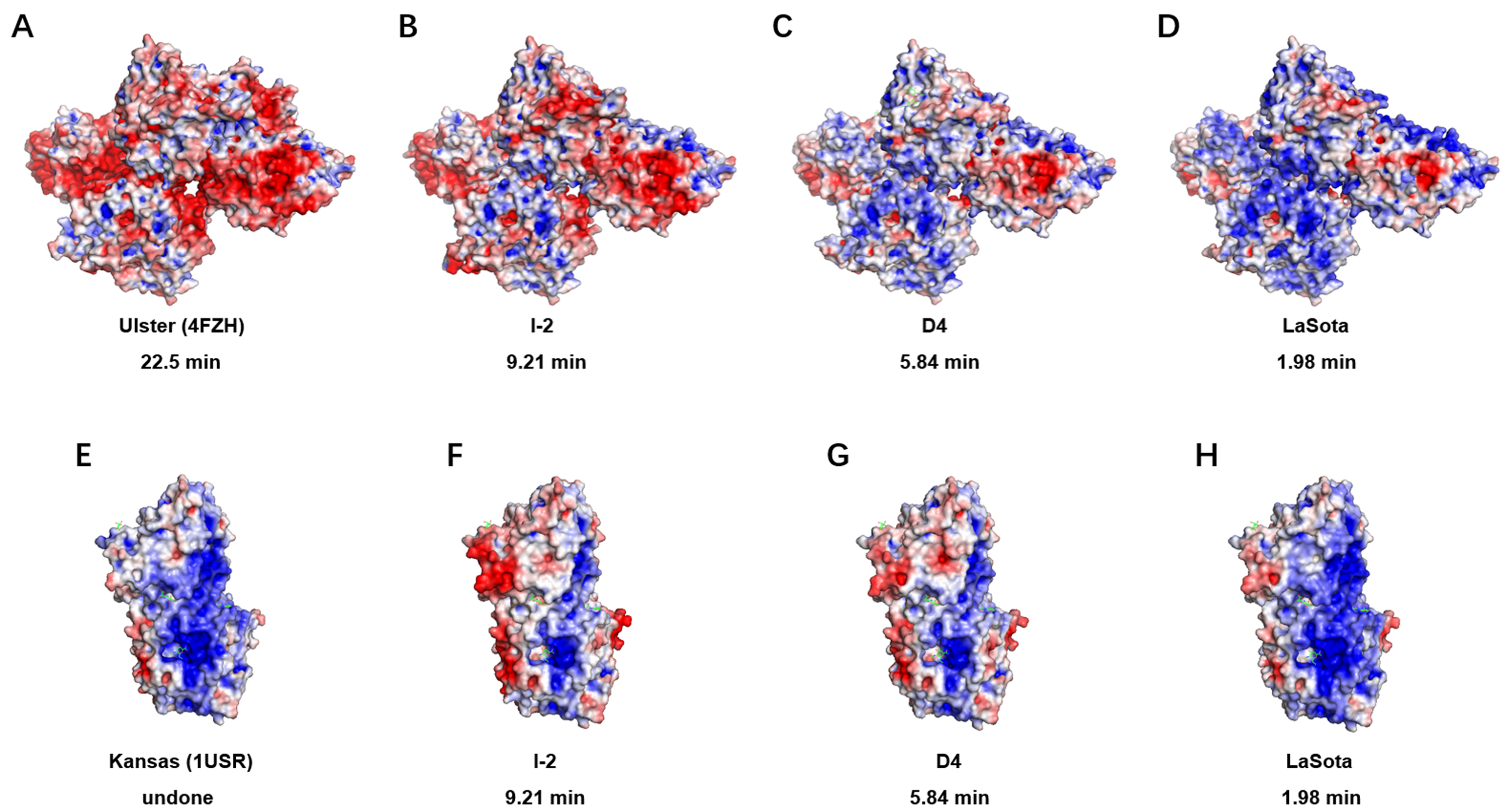

Supplement: S2 Fig — (A-D) The crystal structure of HN protein from Ulster strain (A) is utilized as a template for homology modeling of HN structure of strain I-2, D4, and LaSota. (E-H) The HN structure of Kansas strain (E) is used as a template for homology modeling. The electrostatic potential is mapped to the protein surface as a range of color from red (- 5.0 V) to blue (+ 5.0 V). The names of NDV strains and their corresponding thermostability (Time for 90% infectivity loss, min) at 56°C are indicated at the bottom of structures. The PBD ID codes of HN structures from strain AV and Kansas are indicated in the brackets. (TIF) [file ppat.1010564.s002.tif]

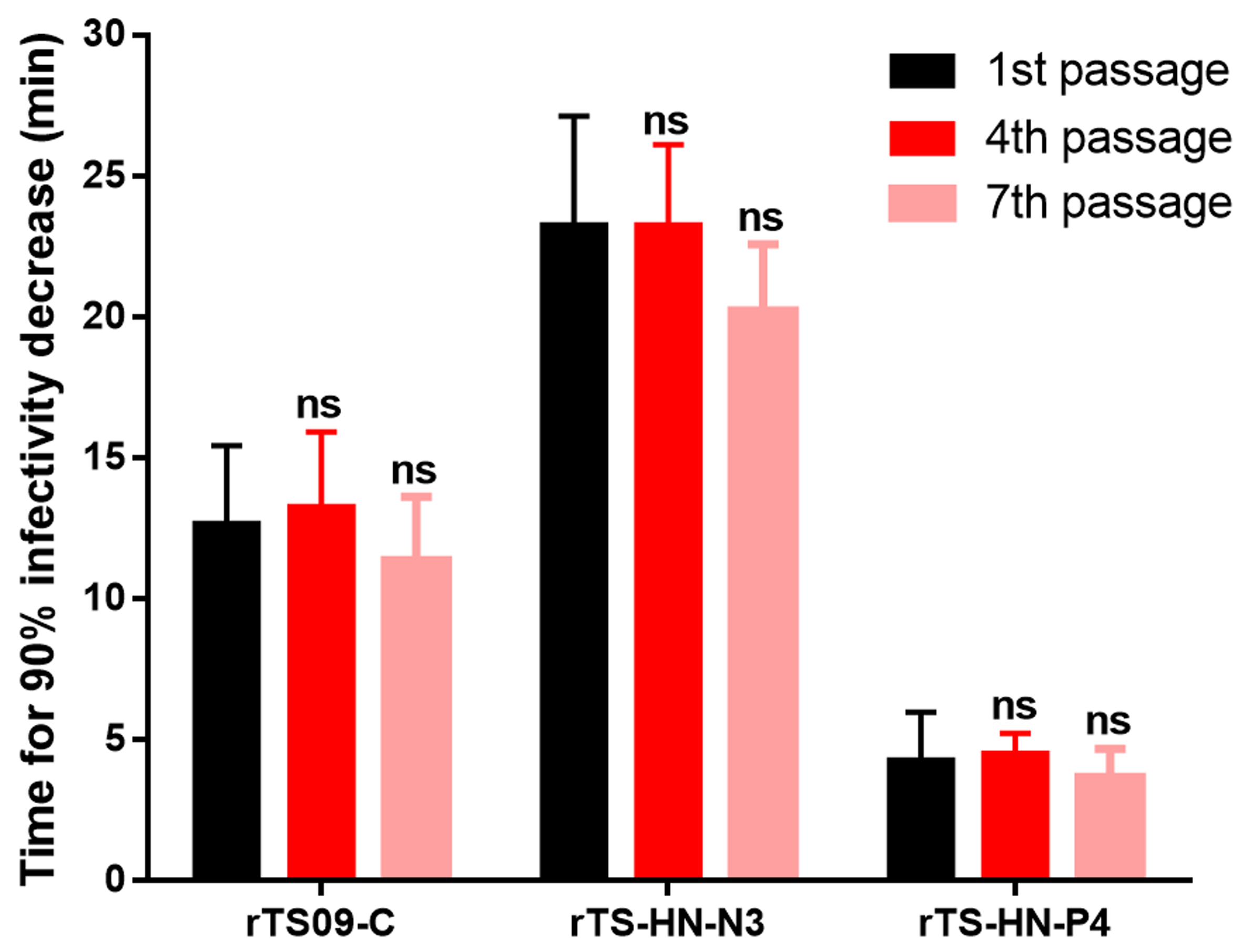

Supplement: S3 Fig — NDV mutants rTS-HN-N3 and rTS-HN-P4, and their parental virus rTS09-C were serially passaged 7 times in chicken embryos, and the thermostability of each virus at 1, 4, and 7 passages was determined at 56°C. (TIF) [file ppat.1010564.s003.tif]

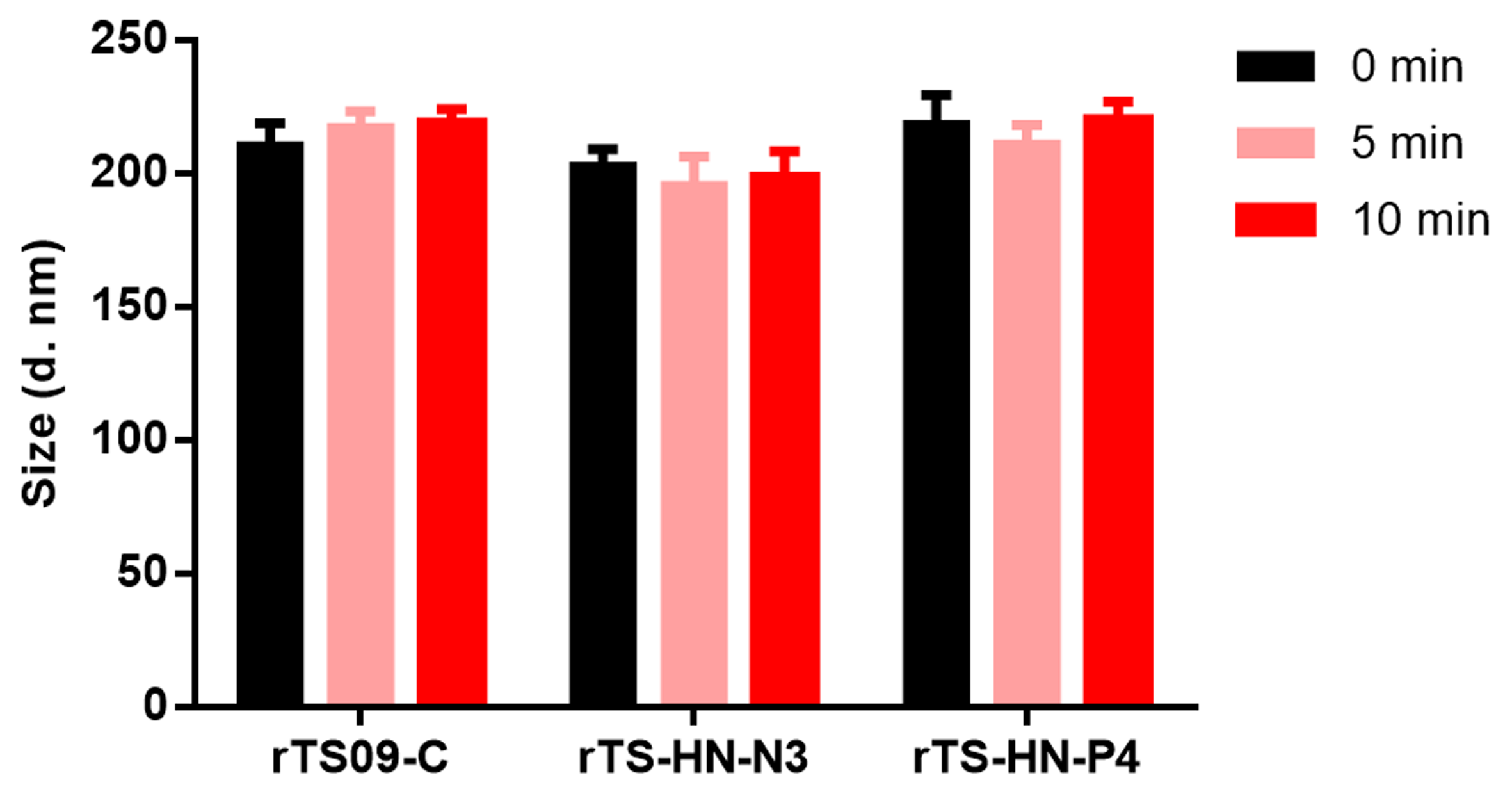

Supplement: S4 Fig — NDV mutants are heat-treated at 56°C for the indicated time, then the sizes of virion are measured using Zetasizer Nano ZS (Malvern). (TIF) [file ppat.1010564.s004.tif]

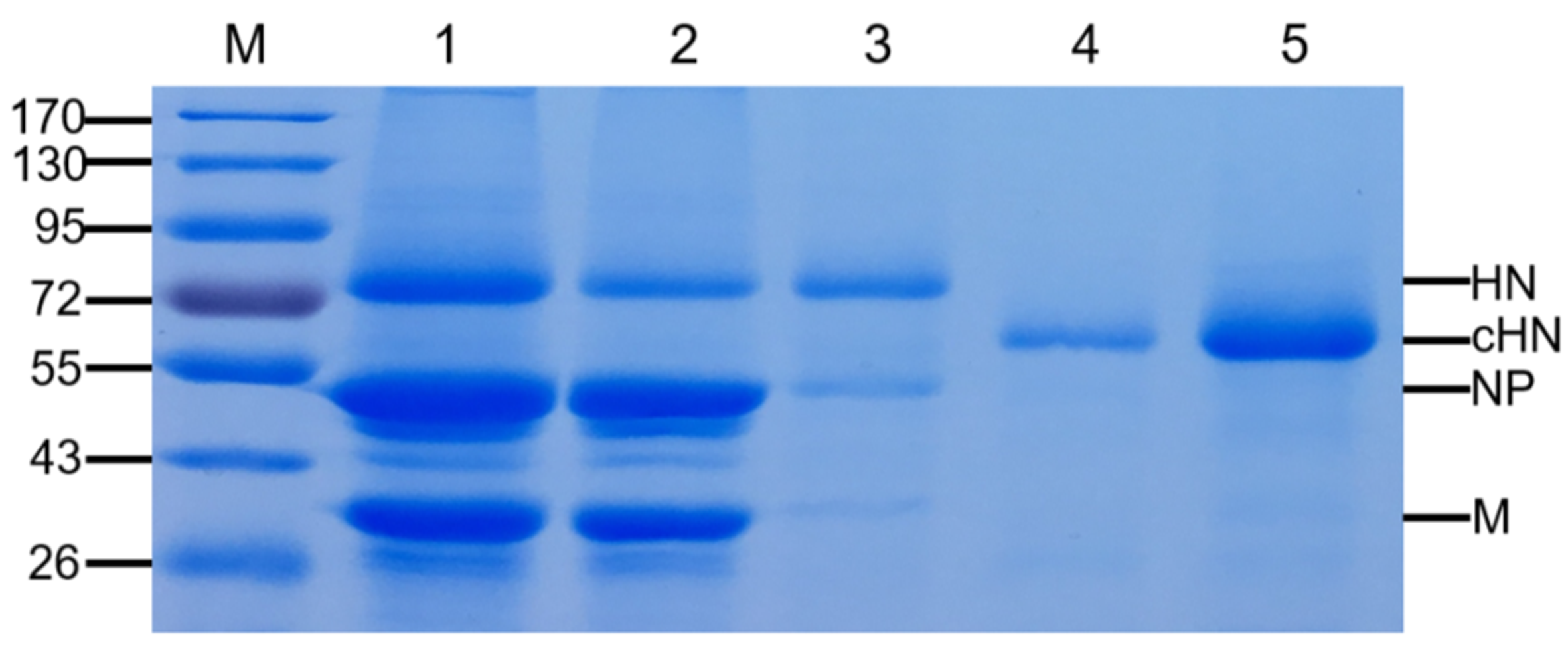

Supplement: S5 Fig — M, protein marker; lane 1, purified NDV; lane 2, NDV treated with Triton X-100; lane 3, reconstituted virosome; lane 4, virosome treated with chymotrypsin; lane 5, purified cHN protein. (TIF) [file ppat.1010564.s005.tif]

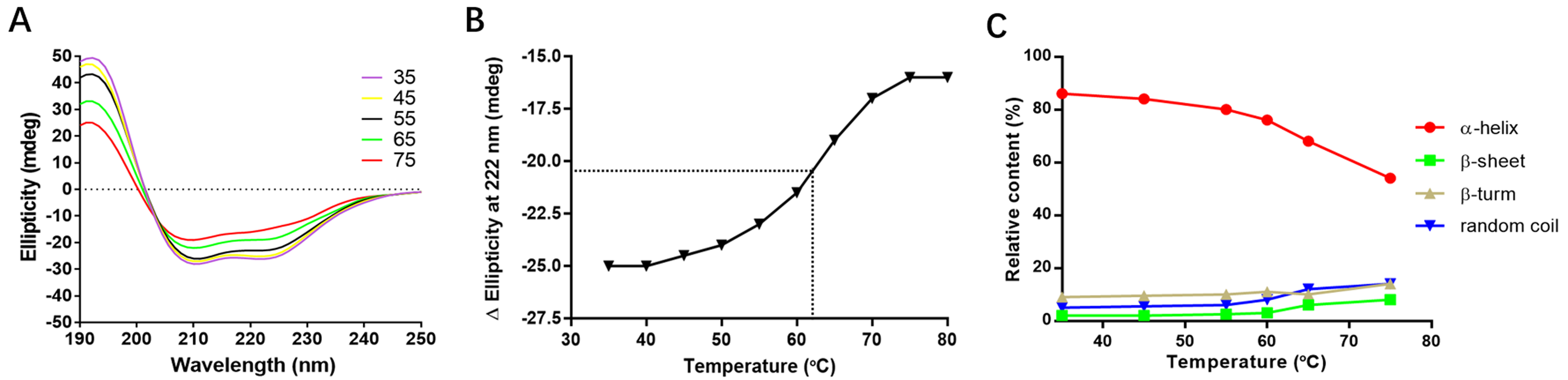

Supplement: S6 Fig — (A) CD spectra of BSA at different temperature ranging from 35°C (purple) to 75°C (red) were measured using CD spectrophotometer J-1500 (JASCO). The legend on the right shows the line colors and their corresponding temperatures. (B) Temperature-induced transition of BSA as monitored by the changes in ellipticity at 222 nm. (C) Secondary structure contents of BSA under different heat-treatment temperature. (TIF) [file ppat.1010564.s006.tif]

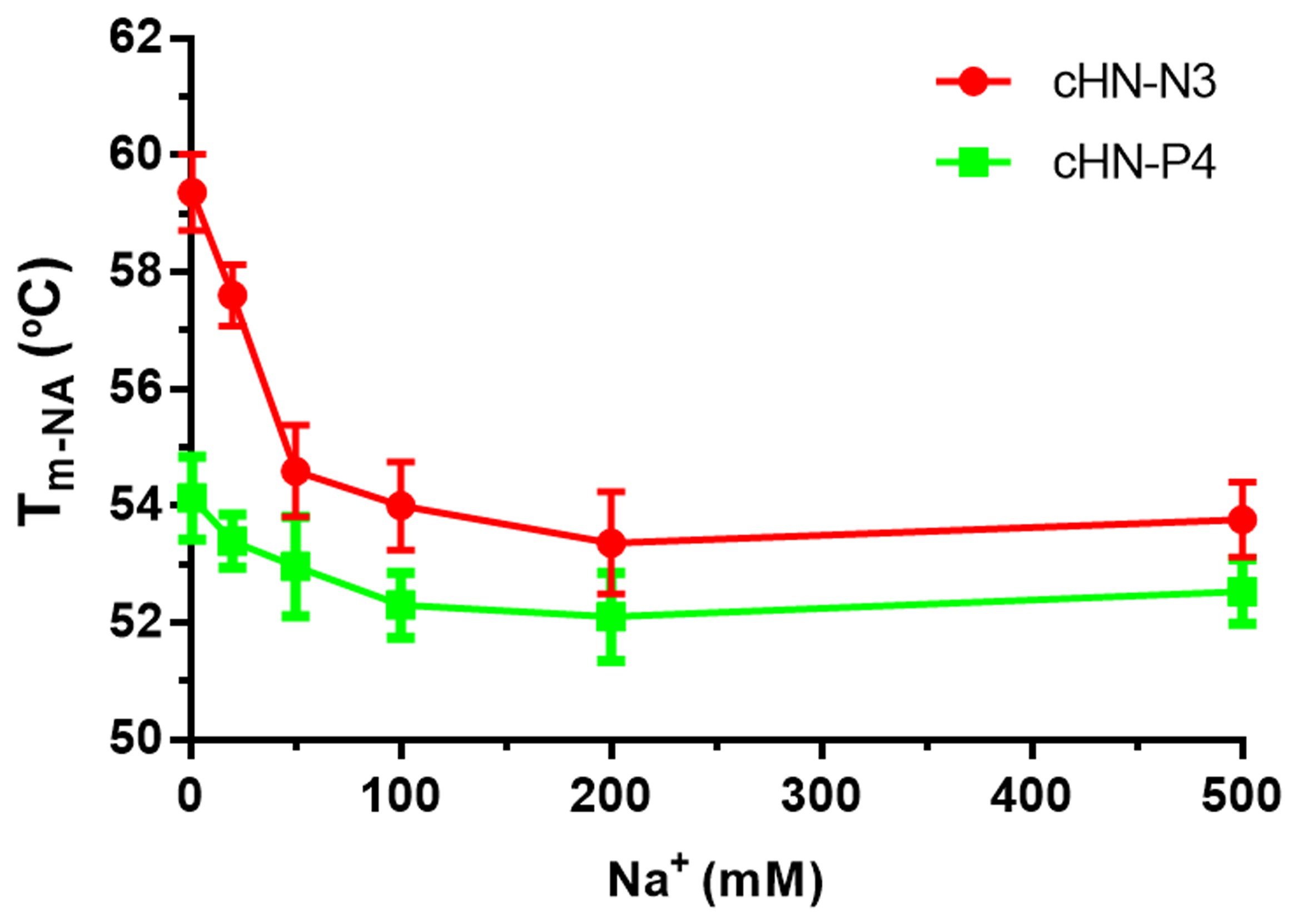

Supplement: S7 Fig — Tm-NA of cHN protein is measured at pH 7.4 and the indicated concentration of NaCl. (TIF) [file ppat.1010564.s007.tif]

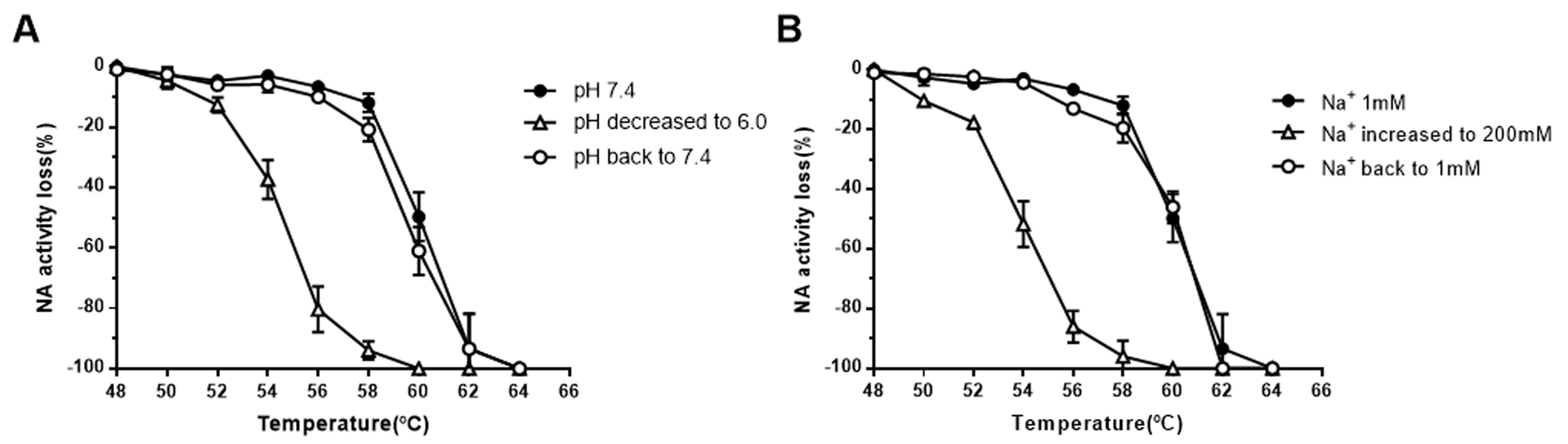

Supplement: S8 Fig — (A) The pH of cHN-N3 solution is adjusted from 7.4 to 6.0 by adding HCl, then back to 7.4 by adding NaOH. After heat-treatment at the indicated temperature for 10 min, the proteins under the three pH conditions (7.4, decreased to 6.0, back to 7.4) are tested for NA activity. (B) The concentration of NaCl in cHN-N3 solution is adjusted from 1.0 to 200mM by adding NaCl, then back to 1.0 mM by filtration. After heat-treatment at the indicated temperature for 10 min, the proteins are tested under three ionic strength conditions (1.0mM, increased to 200mM, back to 1mM) for NA activity. The inactivated fractions of NA activity are represented on a percent scale as a function of heat-treatment temperature. (TIF) [file ppat.1010564.s008.tif]

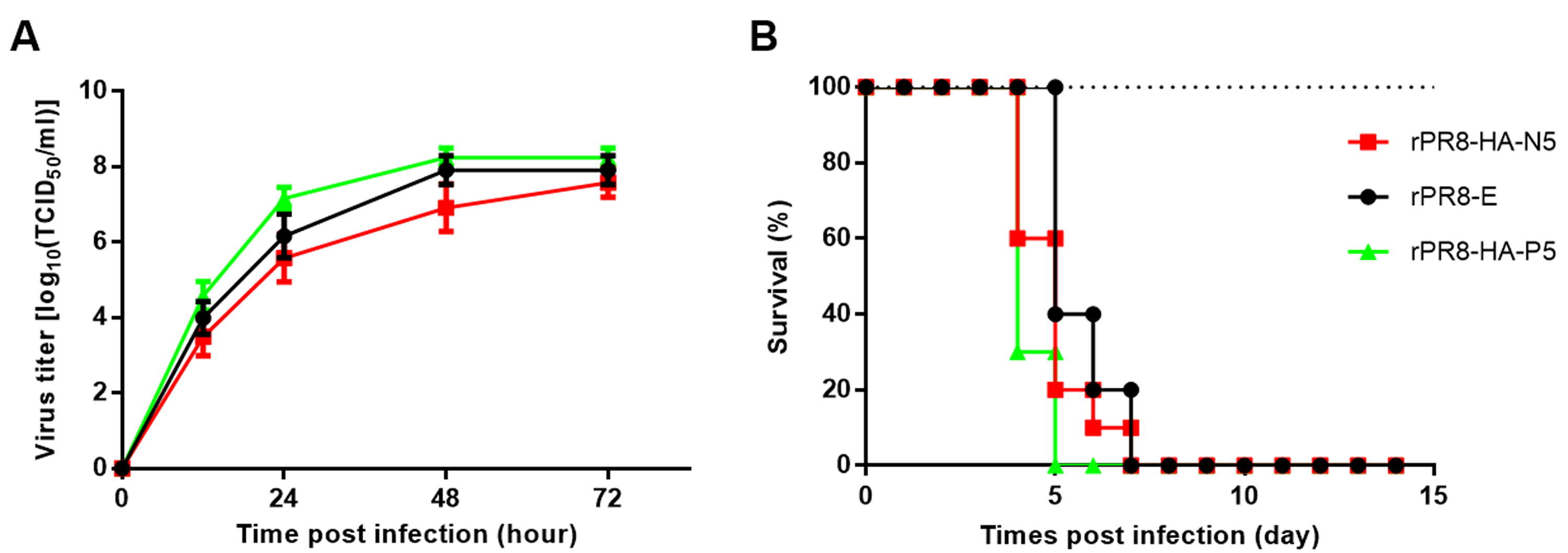

Supplement: S9 Fig — (A) MDCK cells are infected with 0.002 MOI of IAV mutants. At the indicated time points, media from infected cells are collected and titrated for virus yield. (B) BALB/c mice are infected intra-nasally with 103.0 EID50 of IAV mutants, and monitored daily for survival for 14 days. (TIF) [file ppat.1010564.s009.tif]
