# Supplementary material for: Quantitative regulation of the thermal stability of enveloped virus vaccines by surface charge engineering to prevent the self-aggregation of attachment glycoproteins
Source: PLoS Pathog. 2022 Jun 9;18(6):e1010564. doi: 10.1371/journal.ppat.1010564 (PMC9182686; doi:10.1371/journal.ppat.1010564)
Supplement: S1 Table — (DOCX) [file ppat.1010564.s010.docx]

| **Virus** | **Virus titer**  **(Log_10_EID_50_/ml)** | **Pathogenicity** | |
| --- | --- | --- | --- |
|  |  | **MDT (h)** | **ICPI** |
| rTS09-C | 9.47 ± 0.71 | >168 | 0.00 |
| rTS-HN-P2A | 9.35 ± 0.33 | >168 | 0.00 |
| rTS-HN-P2B | 9.57 ± 0.79 | >168 | 0.00 |
| rTS-HN-P4 | 9.26 ± 0.18 | >168 | 0.00 |
| rTS-HN-P11 | 8.90 ± 0.36 | >168 | 0.00 |
| rTS-HN-N3 | 9.18 ± 0.48 | >168 | 0.00 |
| rTS-HN-UN4 | 9.24 ± 0.51 | >168 | 0.00 |
| rTS-HN-PU4 | 9.52 ± 0.72 | >168 | 0.00 |
| rTS-HN-UP4 | 9.16 ± 0.33 | >168 | 0.00 |
| rTS-HN-NU4 | 8.95 ± 0.28 | >168 | 0.00 |
| rLaSota | 9.39 ± 0.41 | 118 | 0.00 |
| rLS-HN-N5 | 9.26 ± 0.81 | >168 | 0.00 |
| rLS-HN-N10 | 8.88 ± 0.39 | >168 | 0.00 |
